# Supplementary figures and images for: H2A.Z deposition by the SWR complex is stimulated by polyadenine DNA sequences in nucleosomes
Source: PLoS Biol. 2025 May 12;23(5):e3003059. doi: 10.1371/journal.pbio.3003059 (PMC12068740; doi:10.1371/journal.pbio.3003059)

# S1 Fig

**A**

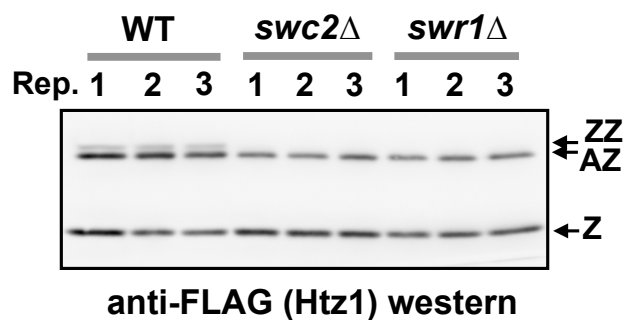

**B**

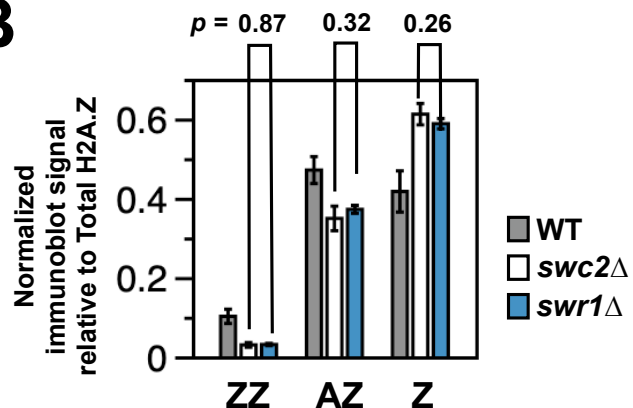

**C**

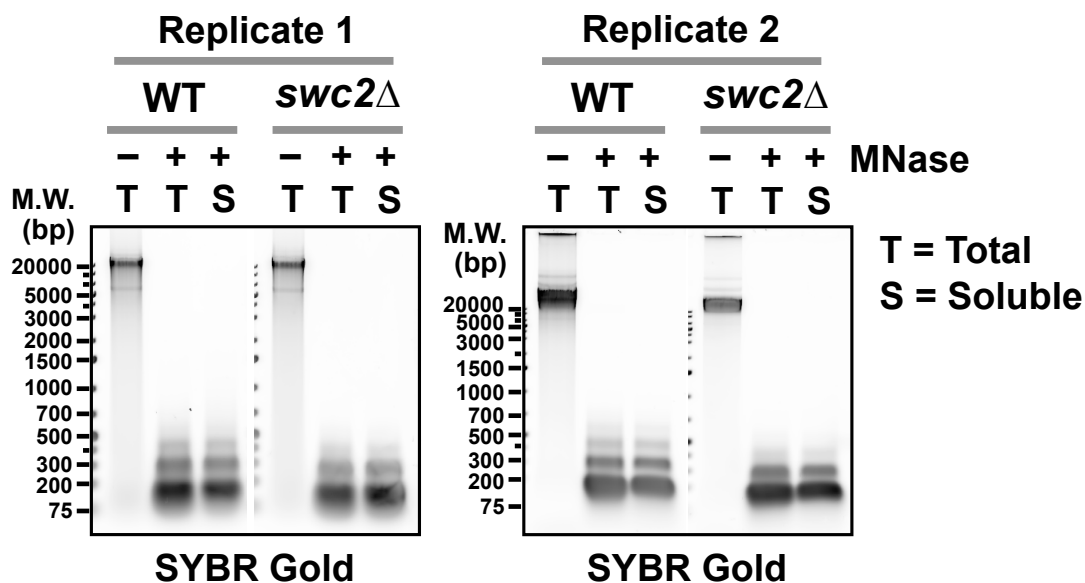

**D**

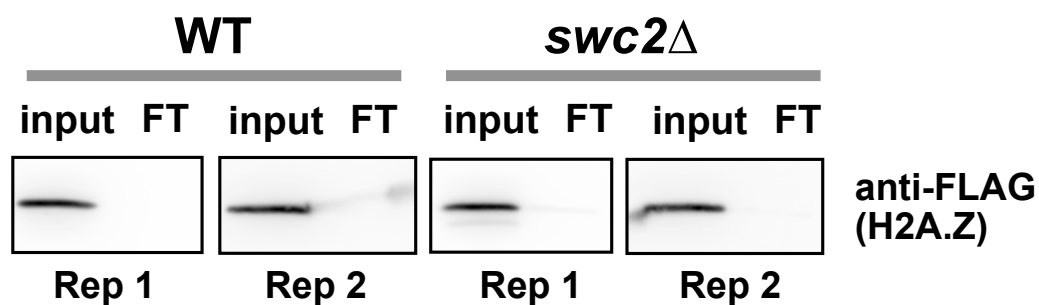

Supplement: S1 Fig — (A) VivosX analysis was performed using WT, swc2Δ and swr1Δ strains bearing the HTZ1(T46C)-2xFLAG and 2xV5-HTA1(N39C) alleles. Three independent cultures (indicated as Rep 1-3) for each strain were grown and analyzed by non-reducing SDS-PAGE and anti-FLAG immunoblotting. (B) Quantification of the immunoblot in A. A t-test was performed to evaluate whether the levels of H2A.Z differed between swr1Δ and swc2Δ. The p-values indicate that there is no significant difference between them. (C) Control for MNase digestion. Chromatin before (-) and after (+) MNase treatment from WT and swc2Δ cells was analyzed by agarose gel electrophoresis and SYBR Gold staining. T: DNA extracted from total extracts. S: DNA extracted from soluble fractions, which were used in the anti-FLAG pulldown reactions against Htz1-2xFLAG. (D) Control for IP efficiency. Equivalent amounts of soluble chromatin before (input) and after immunoprecipitation (FT) were analyzed by SDS-PAGE and anti-FLAG immunoblotting. Rep: replicate. The plot data for S1 Fig B are available in S9 Data. (PDF) [file pbio.3003059.s001.pdf]

# S2 Fig

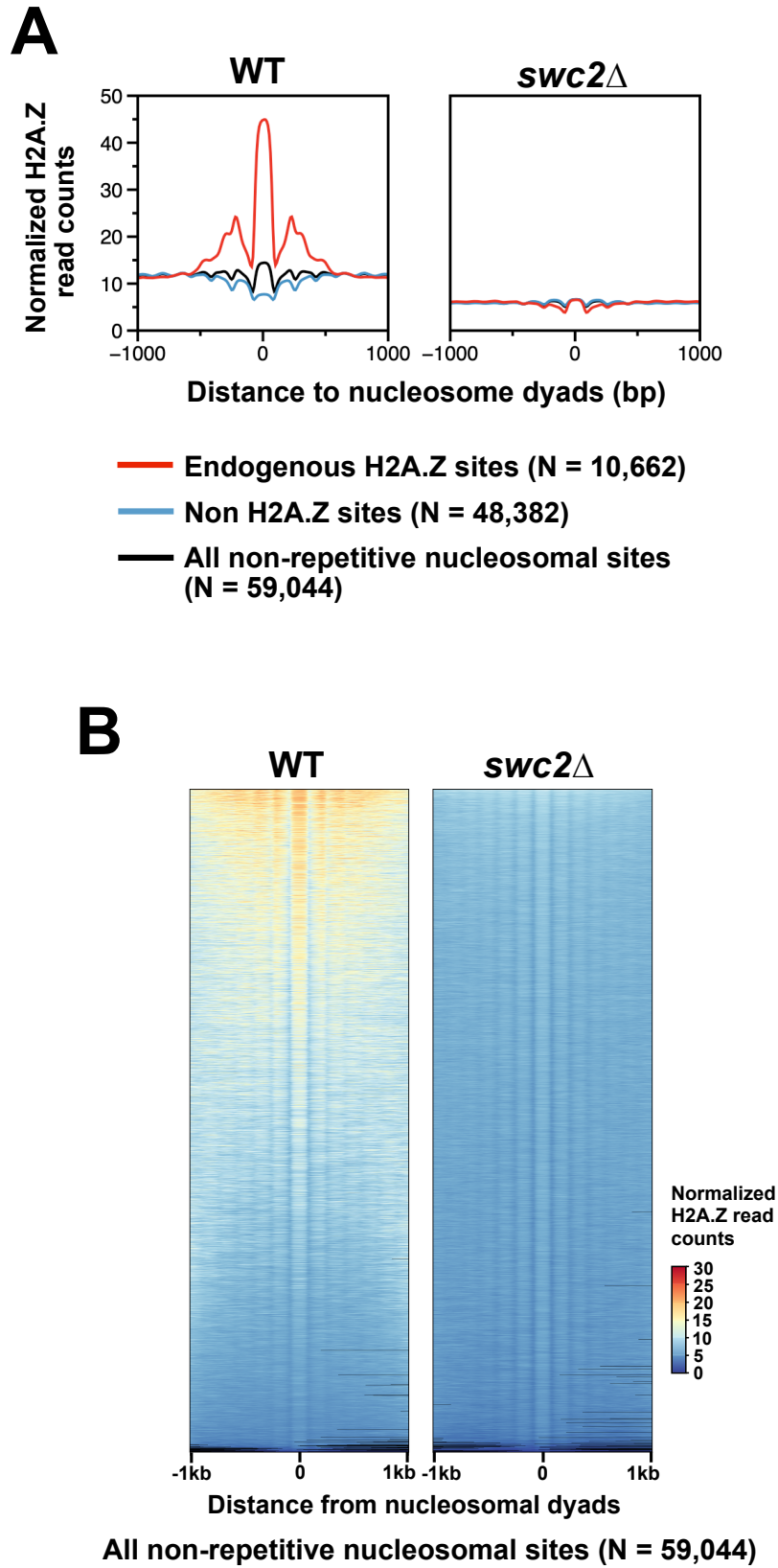

Supplement: S2 Fig — (A) Normalized H2A.Z read counts centered around the dyads of 59,044 nucleosomes in non-repetitive regions (black) for WT (left) and swc2Δ (right) cells. Profiles show nucleosomes grouped by H2A.Z levels; endogenously enriched H2A.Z sites in red and non-H2A.Z sites in blue. (B) Heatmaps show the alignment of these 59,044 non-repetitive nucleosomes around the nucleosomal dyads used in panel A, for both WT and swc2Δcells. The plot data for S2 Fig A are available in S10 Data. The plot data for the heatmaps in S2 Fig B are available in S11 Data (left panel) and S12 Data (right panel) as gzip-compressed text files. (PDF) [file pbio.3003059.s002.pdf]

S3 Fig

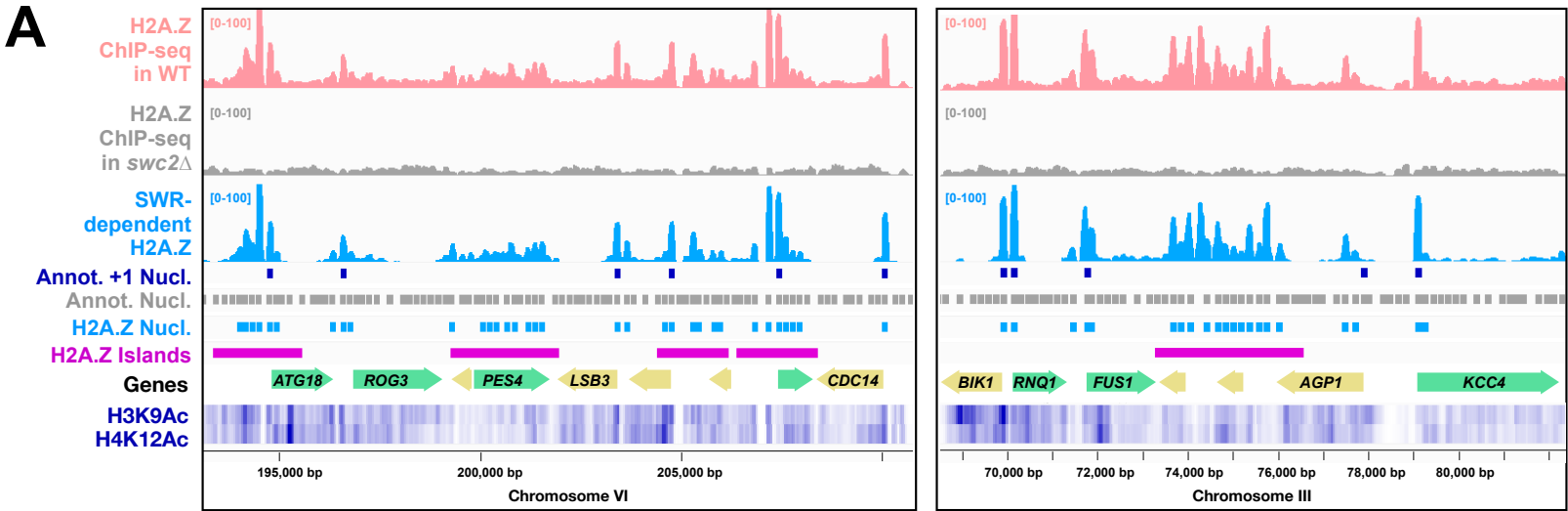

**B** SWR-dependent H2A.Z

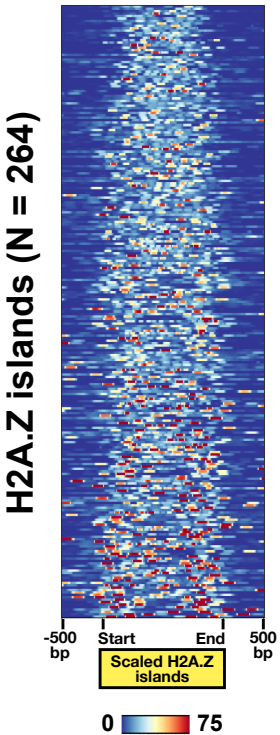

**C**

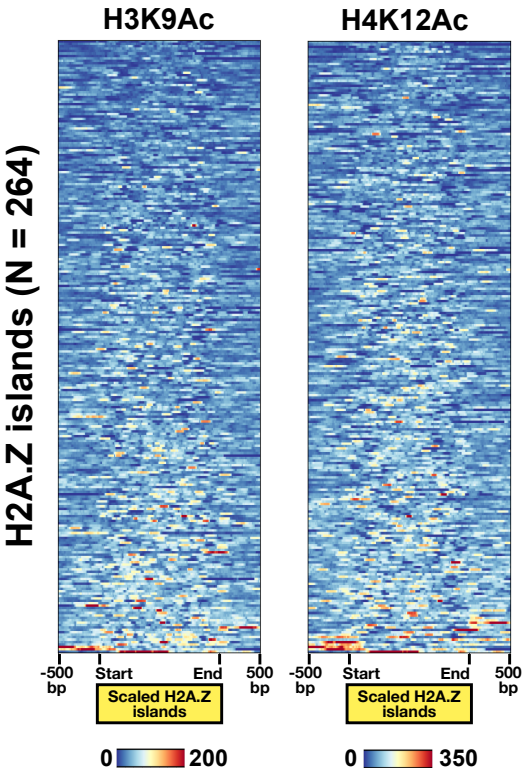

**D**

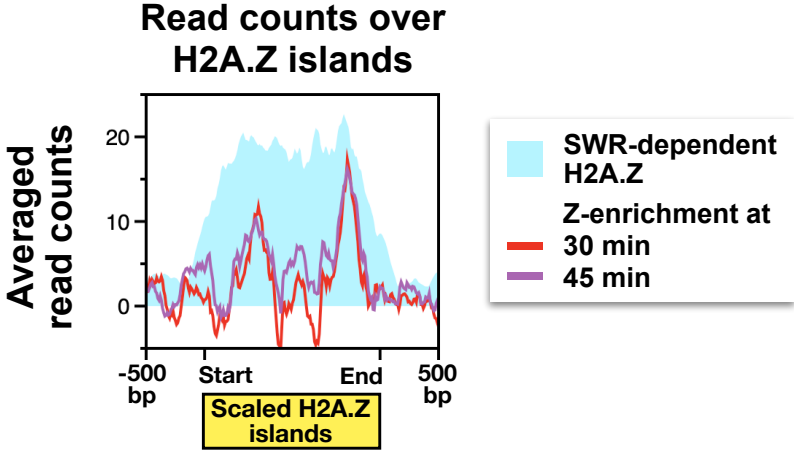

Supplement: S3 Fig — (A) Same as 1D except that two other regions with H2A.Z islands are shown. (B) A heatmap showing SWR-dependent H2A.Z levels of 264 H2A.Z islands. H2A.Z islands were aligned at their starts and ends, scaled to equal length, and sorted by H2A.Z levels. (C) Heatmaps showing H3K9Ac and H4K12Ac levels of the 264 H2A.Z islands. (D) Averaged SWR-dependent H2A.Z (endogenous) along 264 H2A.Z islands were compared to Z-enrichment scores representing SWR’s preference in vitro. The track information for S3 Fig A and plot data for S3 Fig B–D are available in S13 Data. (PDF) [file pbio.3003059.s003.pdf]

S4 Fig

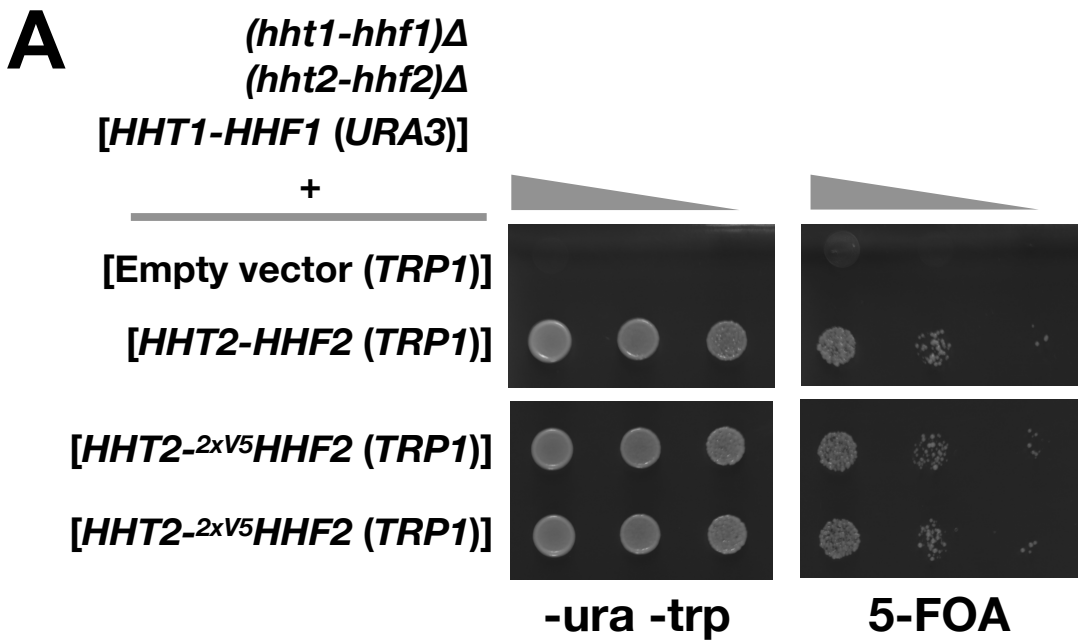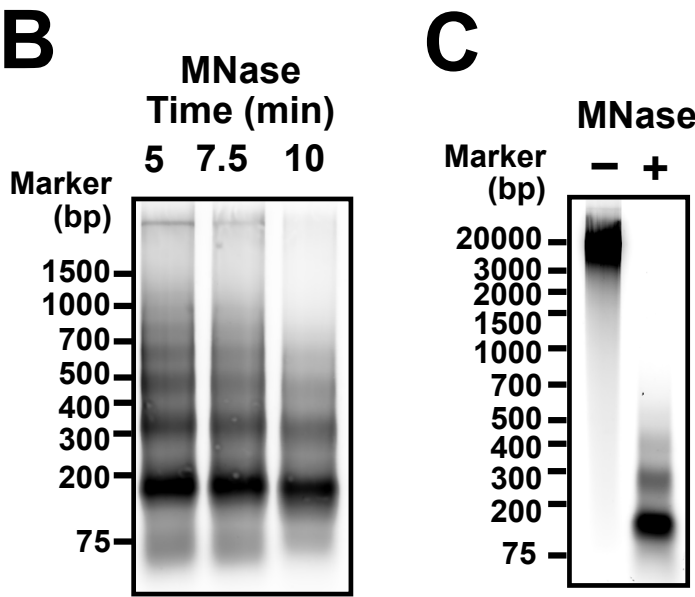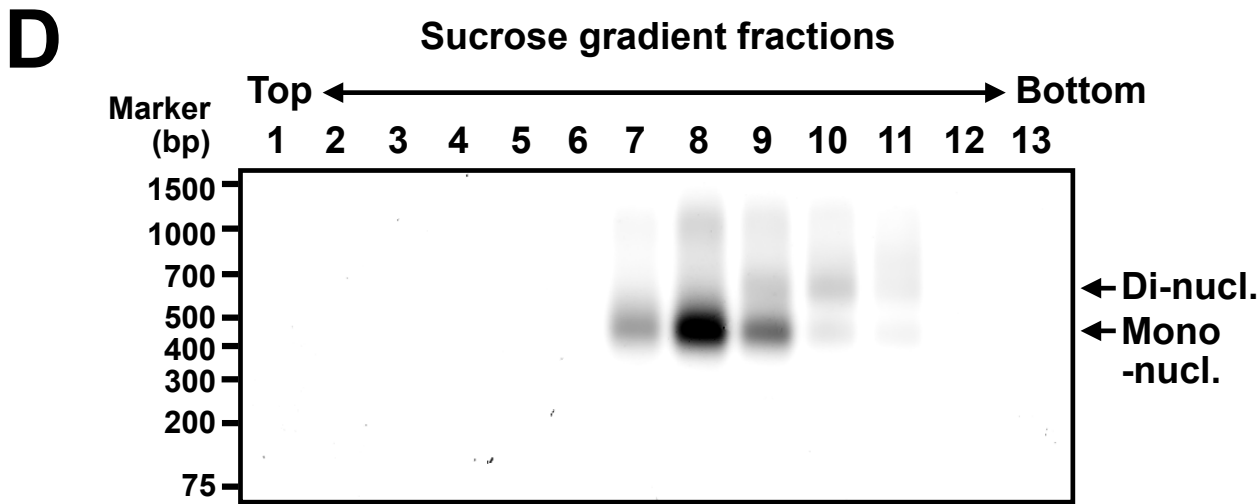

Supplement: S4 Fig — (A) Complementation test showing that the 2xV5-HHF2 gene is functional. The HHT2-(2xV5-HHF2) TRP1 CEN ARS plasmid, the untagged control or the empty vector were transformed into a yeast strain that lacked the endogenous H3 and H4 genes but was kept alive by a wild-type HHT1-HHF1 URA3 CEN ARS plasmid. Ten-fold serially diluted cells (starting at 1 OD600) were spotted onto synthetic complete media lacking uracil and tryptophan (left) or media supplemented with 5-FOA (right). (B) Optimization of MNase digestion. Yeast chromatin was incubated with MNase for the indicated times before extracted for DNA analysis. (C) Scale-up of MNase digestion before (–) and after (+) MNase treatment. (D) Nucleosomes containing V5-tagged H4 were affinity purified using anti-V5 agarose and sedimented through a 15-40% sucrose gradient. The fractions were analyzed by 1.3% agarose / 0.5x TBE electrophoresis and SYBR gold staining. (PDF) [file pbio.3003059.s004.pdf]

S6 Fig

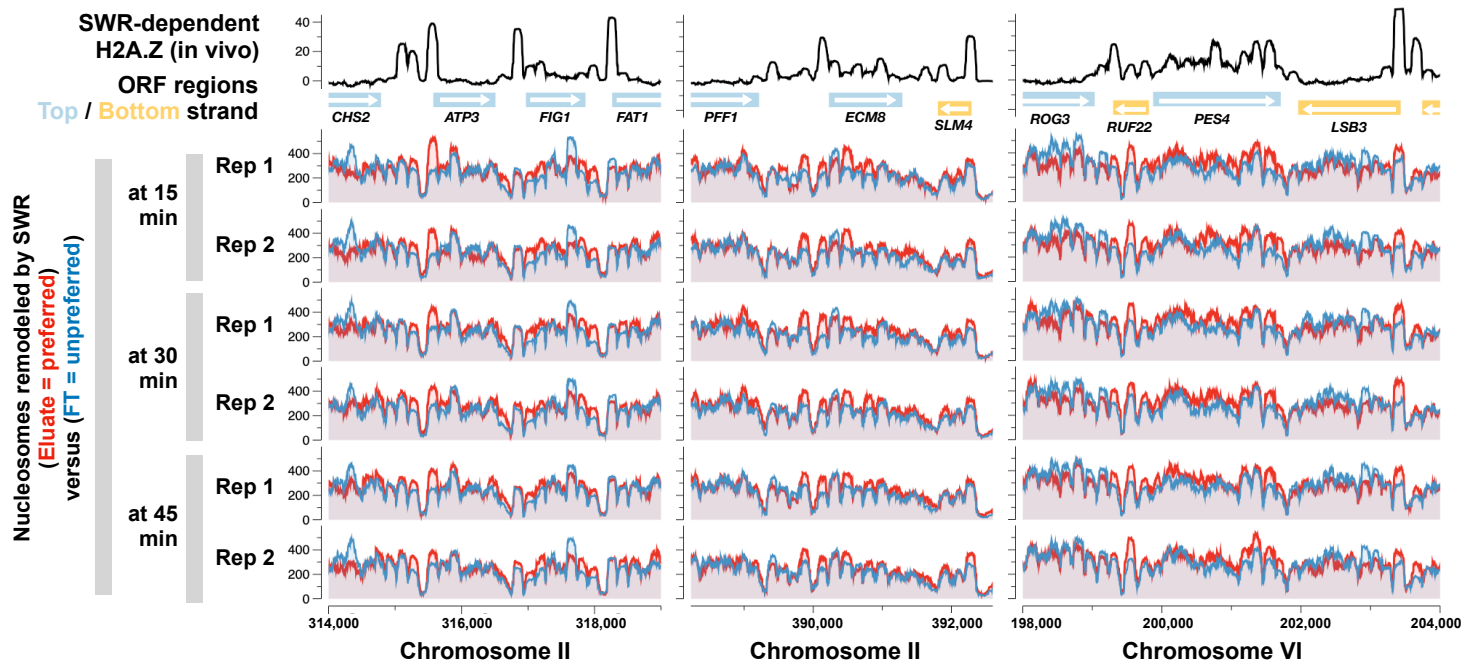

Supplement: S6 Fig — Sequencing read coverages of the nucleosomes in the eluate (SWR-preferred) and FT (unpreferred) fractions of the streptavidin pulldown after histone exchange reactions. Red: eluate. Blue: FT. Black traces: endogenous H2A.Z. Three representative regions are shown. The plot data for S6 Fig are available in S14 Data. (PDF) [file pbio.3003059.s006.pdf]

# S7 Fig

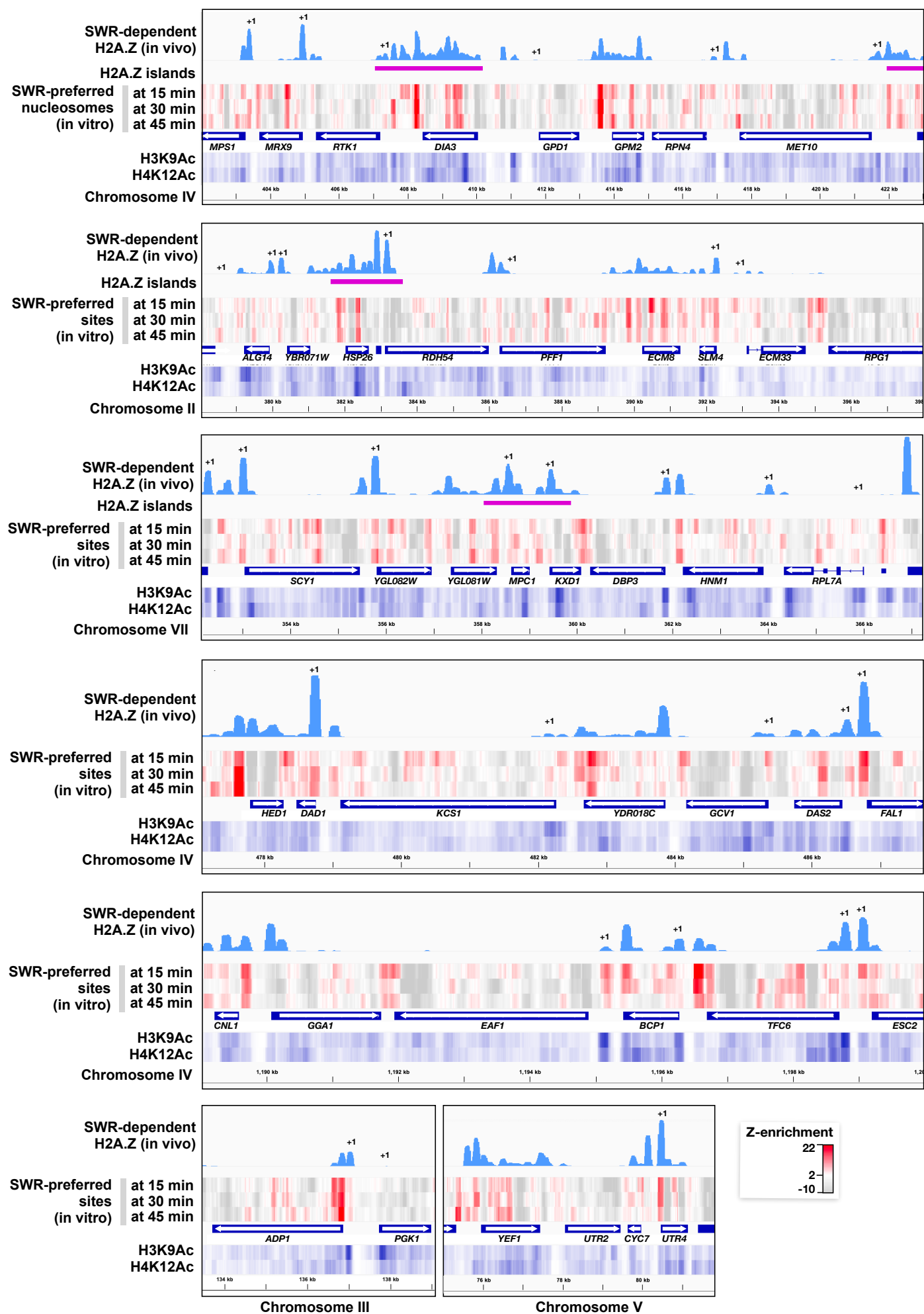

Supplement: S7 Fig — Same as Fig 3C, with additional regions included in the plot. The track information for S7 Fig is available in S15 Data. (PDF) [file pbio.3003059.s007.pdf]

S8 Fig

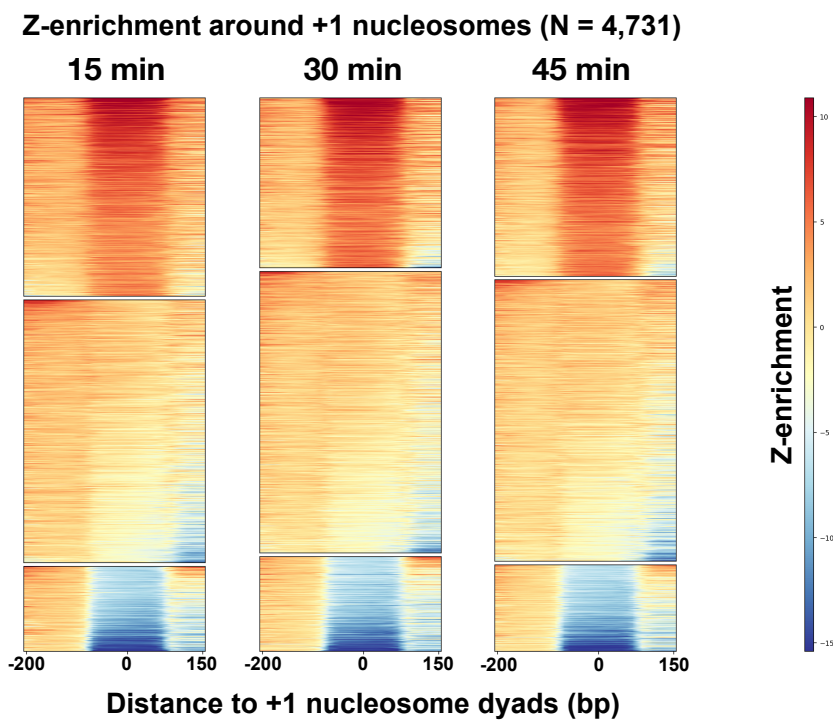

Supplement: S8 Fig — Z-enrichment values were plotted around 4,731 annotated +1 nucleosomes. Their profiles were analyzed by k-means cluster (k = 3). The resulting +1 nucleosome list can be found in S1 Data. The plot data are available in S16 Data. (PDF) [file pbio.3003059.s008.pdf]

**S9 Fig****A**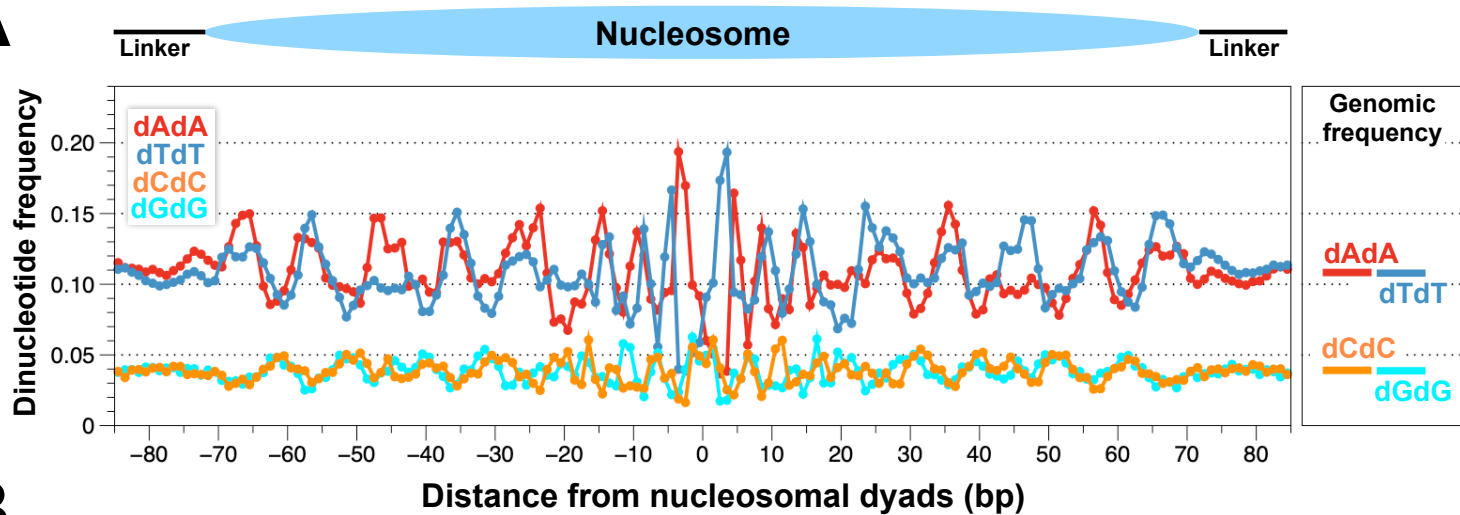**B**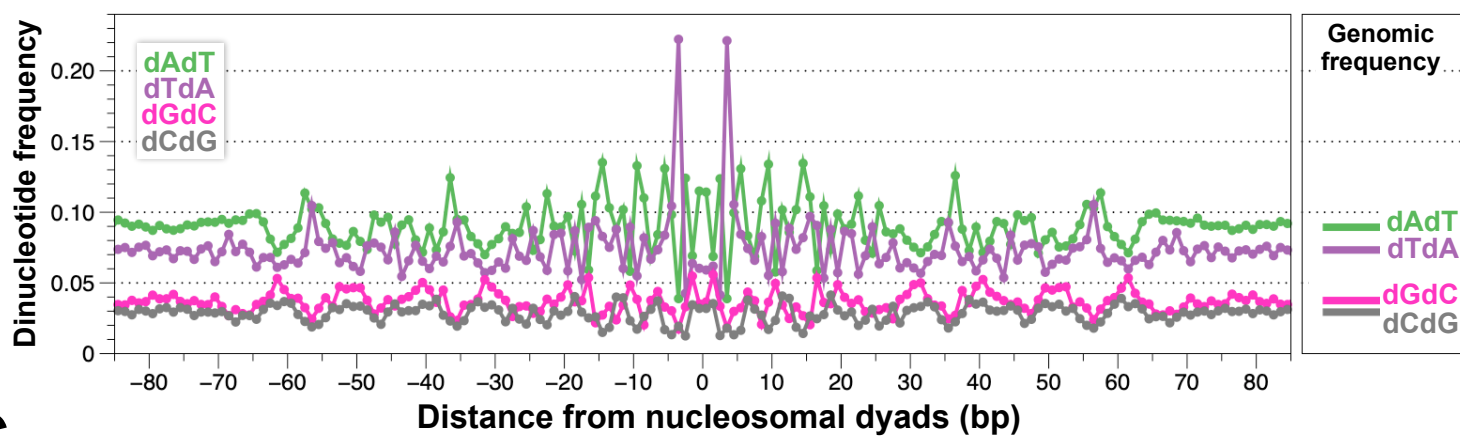**C**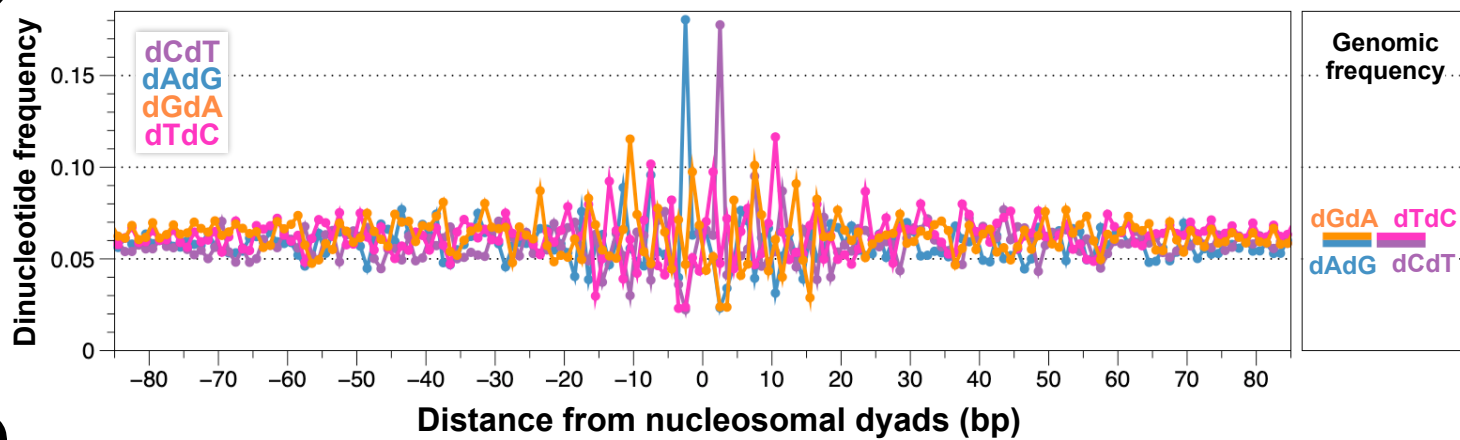**D**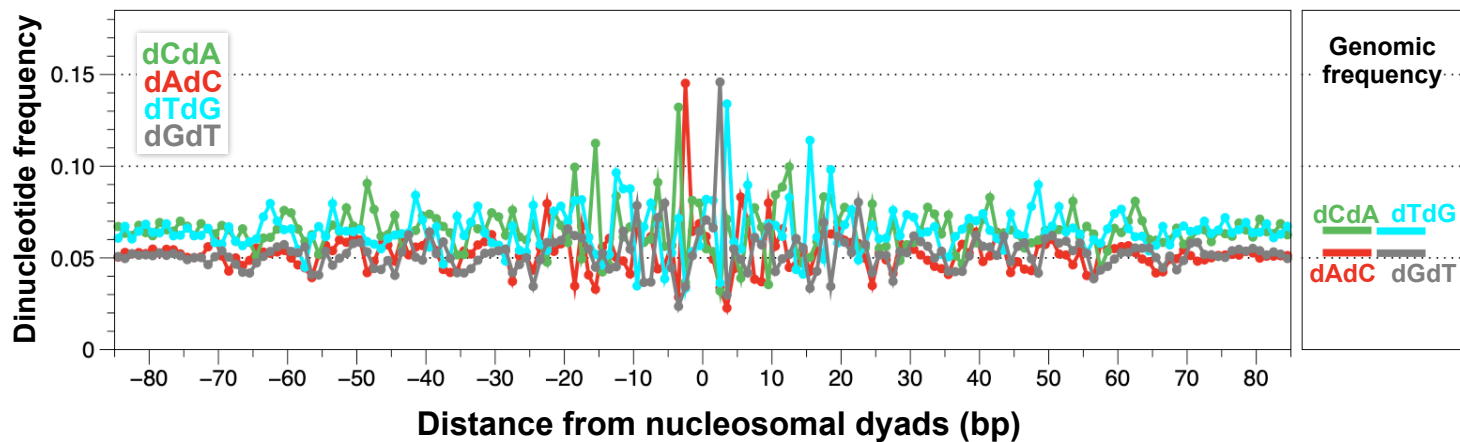

Supplement: S9 Fig — (A) Dinucleotide frequencies of dAdA, dTdT, dCdC, and dGdG plotted along nucleosome positioning sequences (N = 67,538) centered at nucleosomal dyads plus 20 bp of flanking regions. The box on the right shows the frequencies of the dinucleotides across the genome. (B-D) Same as A, except showing the indicated dinucleotide frequencies. The plot data for S9 Fig are available in S17 Data. (PDF) [file pbio.3003059.s009.pdf]

S10 Fig

SWR-preferred nucleosomes

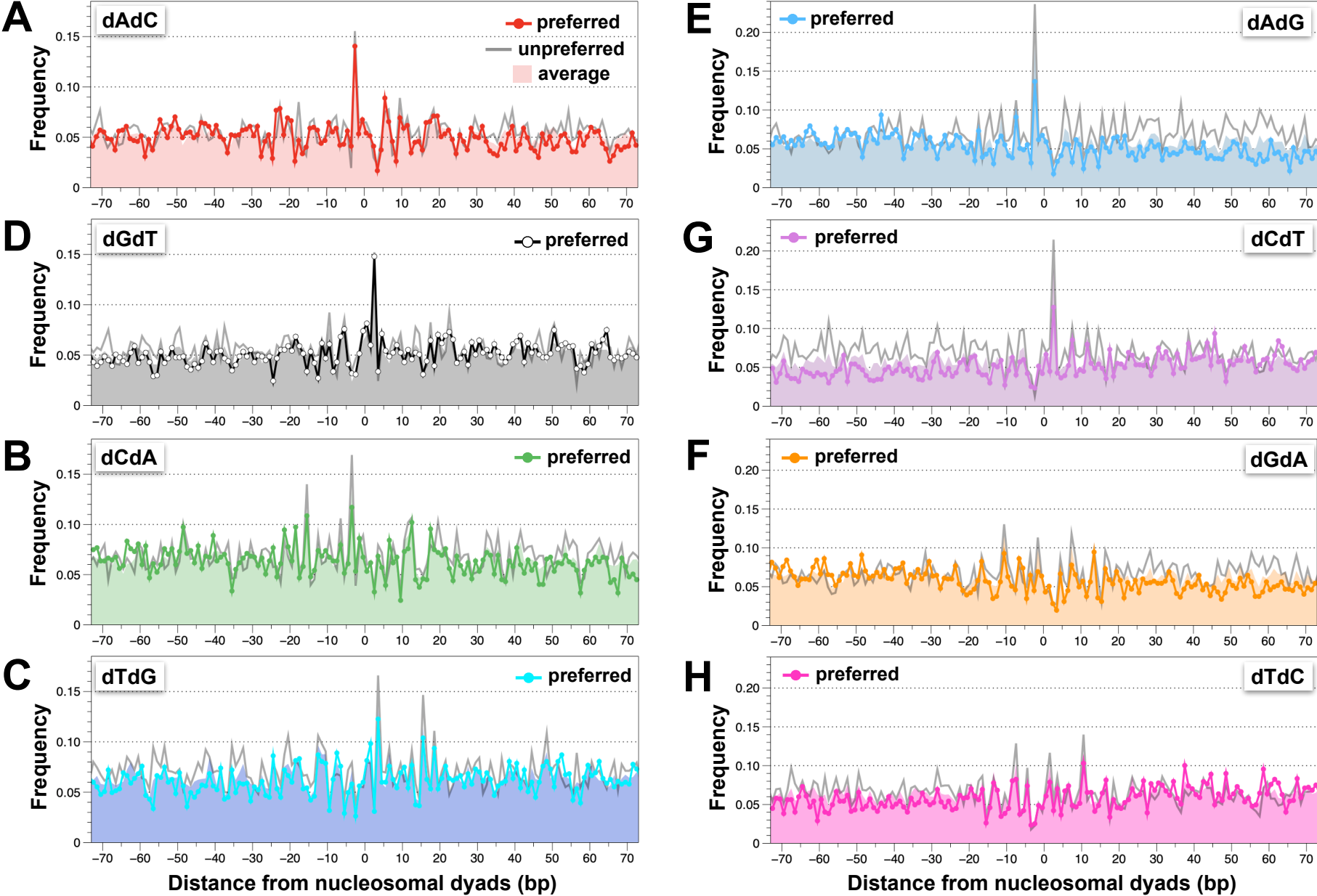

Supplement: S10 Fig — (A–H) Same as Fig 5, except showing other dinucleotides. The plot data for S10 Fig are available in S18 Data. (PDF) [file pbio.3003059.s010.pdf]

S11 Fig

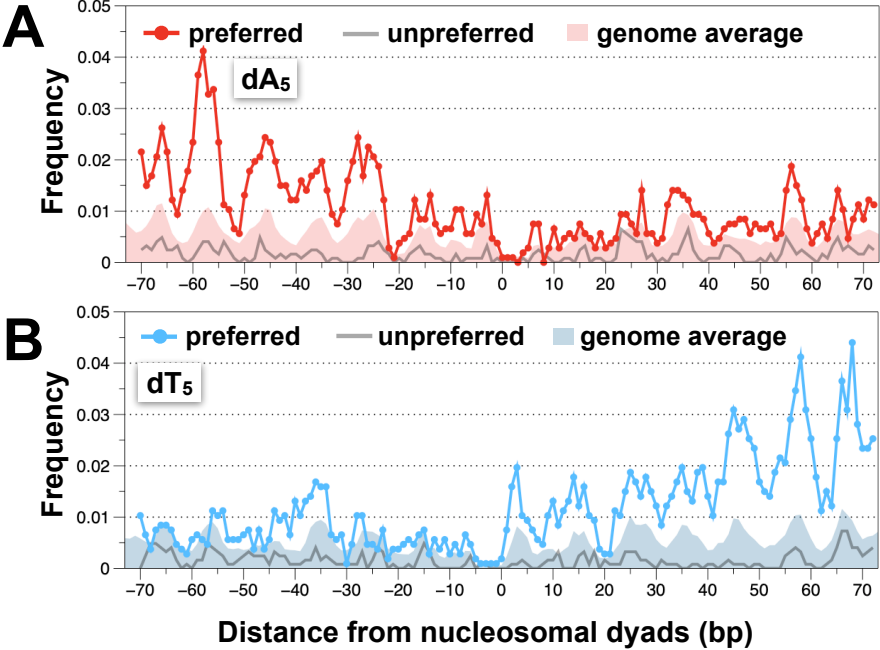

Supplement: S11 Fig — (A–B) Same as Fig 5, except showing the frequency of five consecutive dA (dA5) and dT (dT5). The plot data for S11 Fig are available in S19 Data. (PDF) [file pbio.3003059.s011.pdf]

S12 Fig

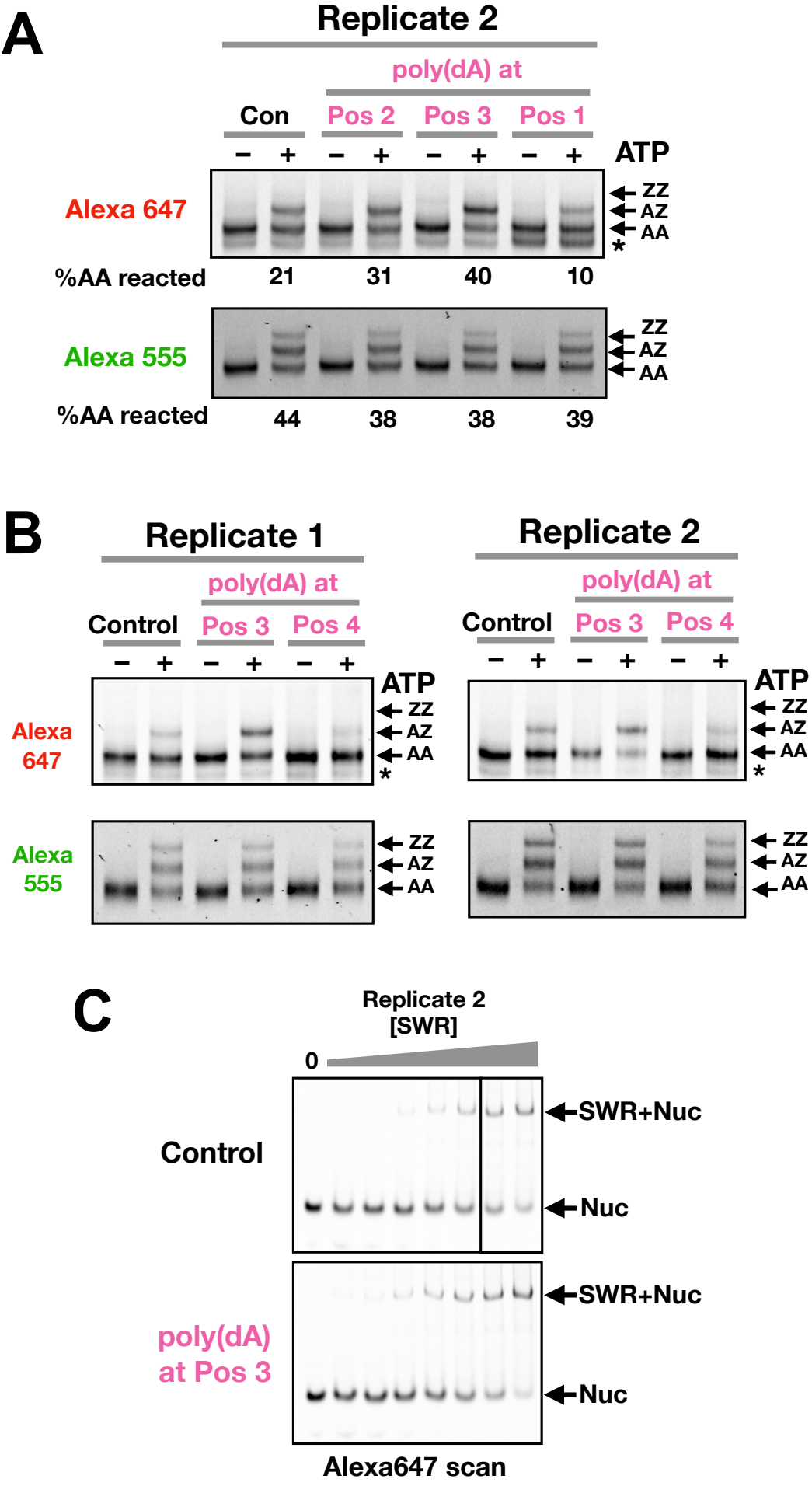

Supplement: S12 Fig — (A) A replicate of the experiment in Fig 6D. (B) Same as A, except including a nucleosomal substrate with a poly(dA) tract at Pos 4. (C) A replicate of the binding experiment in Fig 6E. (PDF) [file pbio.3003059.s012.pdf]

S13 Fig

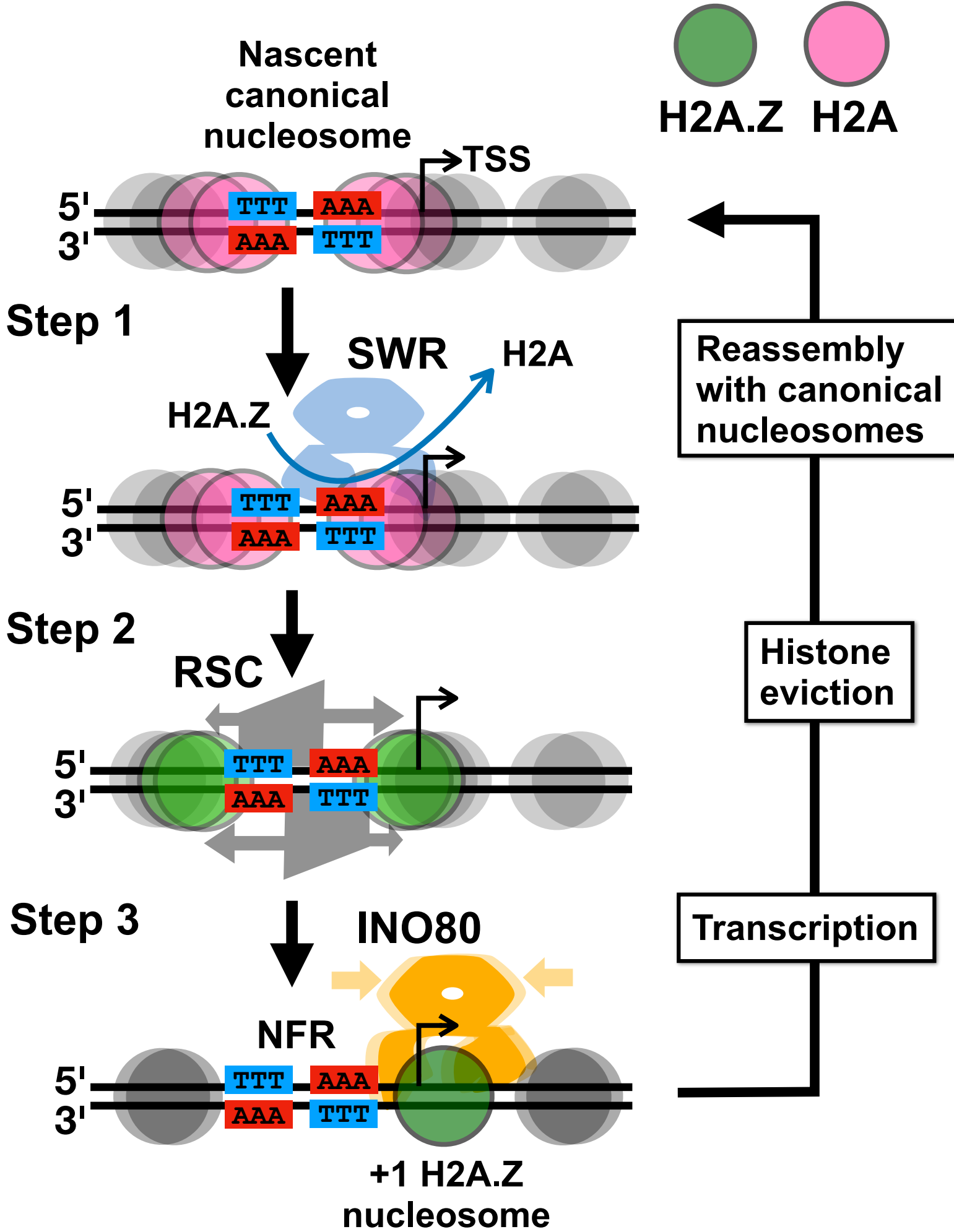

Supplement: S13 Fig — Pink circles: NDR-proximal nucleosomes containing H2A. Green circles: NDR-proximal nucleosomes containing H2A.Z. Grey circles: NDR-distal nucleosomes. AAA and TTT indicates poly(dA) and poly(dT) tracts respectively. Blue crab: SWR complex. Orange crab: INO80 complex. Trapezoid: RSC complex. (PDF) [file pbio.3003059.s013.pdf]

# S14 Fig

**A**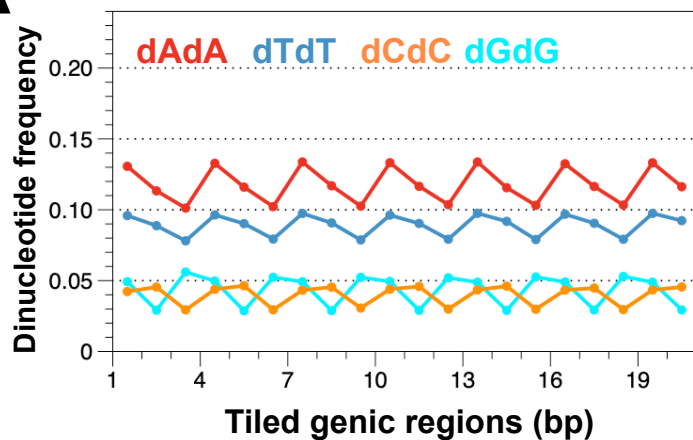**B**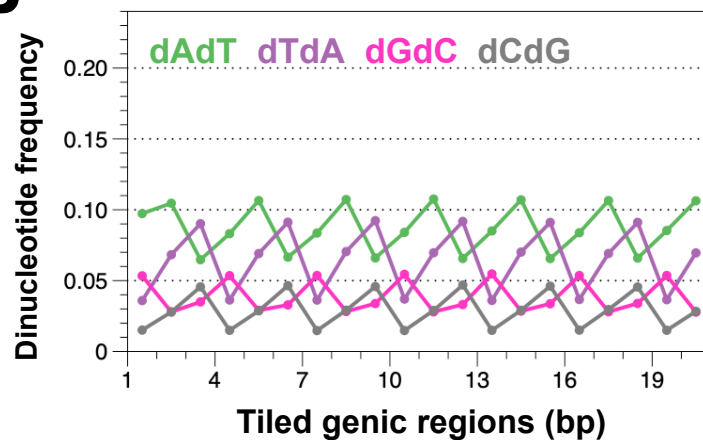**C**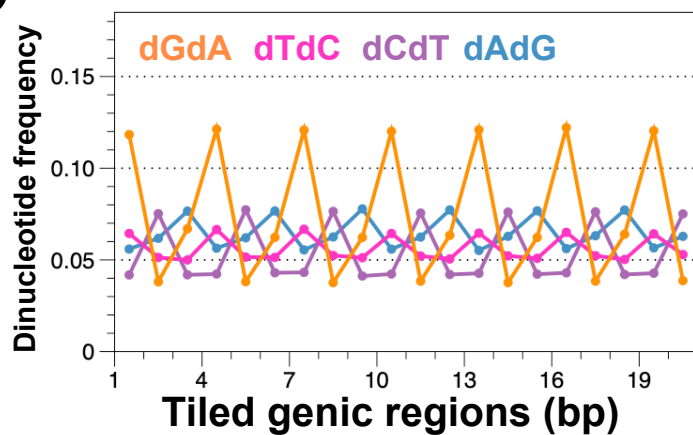**D**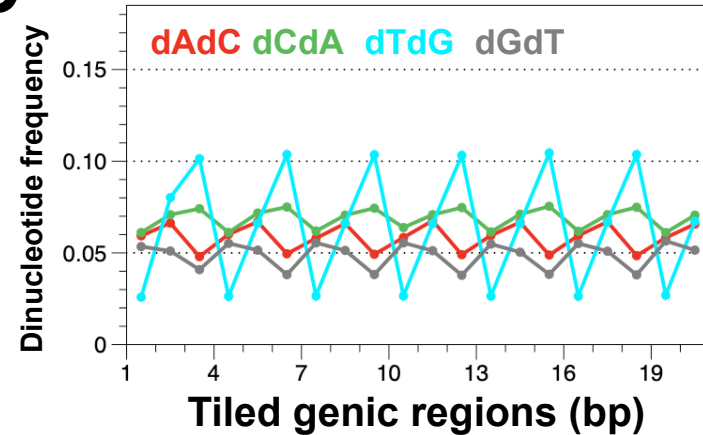

Supplement: S14 Fig — (A–D) The coding regions of 6,401 yeast genes were divided into 21-bp tiled fragments in-frame with the genetic codes. The indicated dinucleotide frequencies were averaged over 422,795 tiled regions. The plot data for S14 Fig are available in S20 Data. (PDF) [file pbio.3003059.s014.pdf]

**S15 Fig**

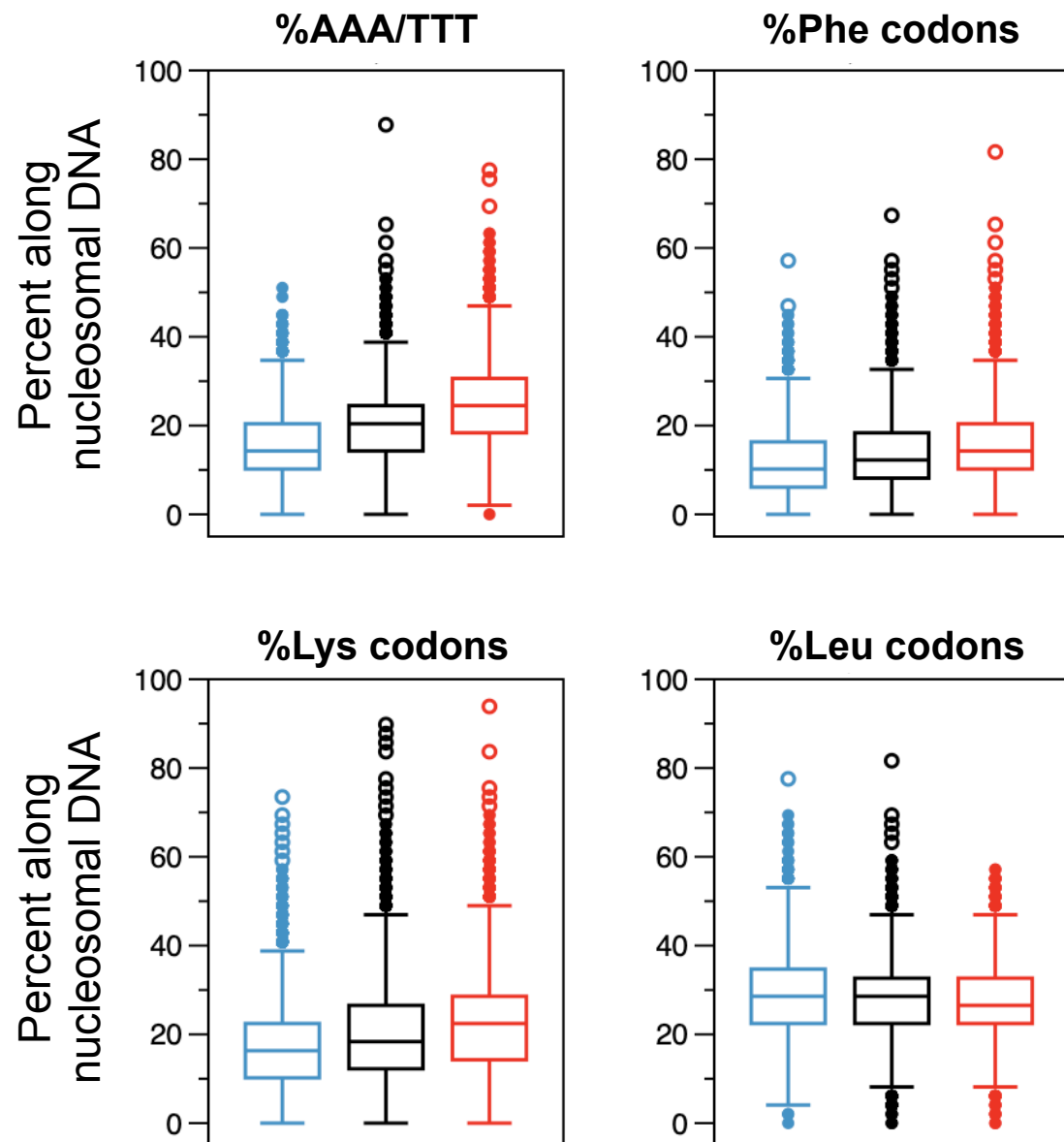

— Top 25% Z-enrichment at 30 min (N = 9983)  
— Middle 50% Z-enrichment at 30 min (N = 19967)  
— Bottom 25% Z-enrichment at 30 min (N = 9984)

Supplement: S15 Fig — The plot data for S15 Fig are available in S21 Data. (PDF) [file pbio.3003059.s015.pdf]

S16 Fig

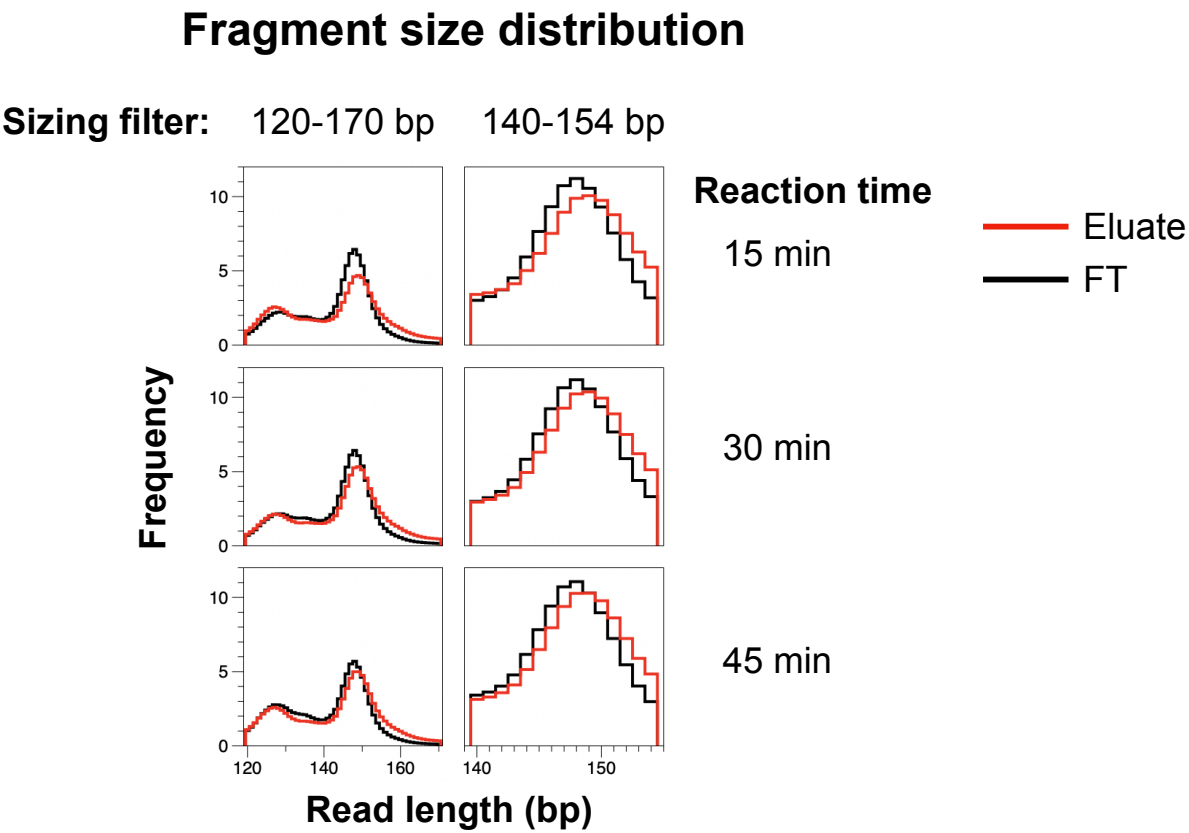

Supplement: S16 Fig — Read length distribution of the nucleosomal DNA in the eluate and FT fractions of the in vitro SWR-mediated reactions were analyzed after applying the indicated sizing filters. The plot data for S16 Fig are available in S22 Data. (PDF) [file pbio.3003059.s016.pdf]

S17 Fig

**A**

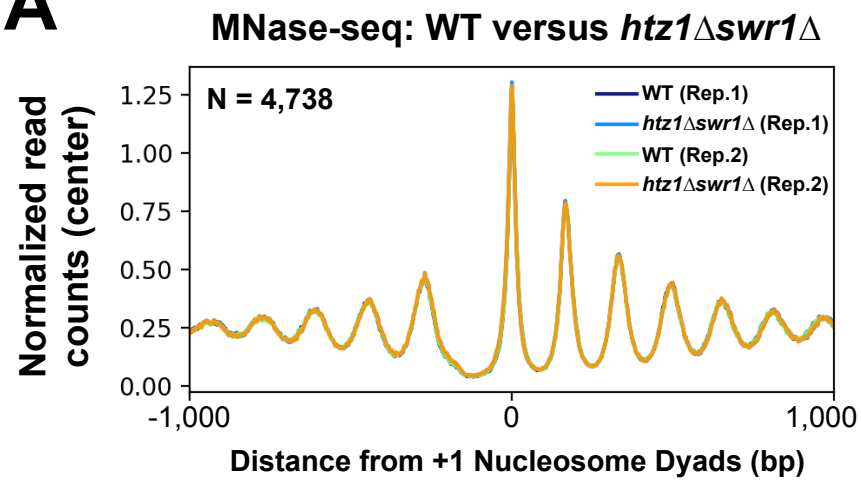

**B**

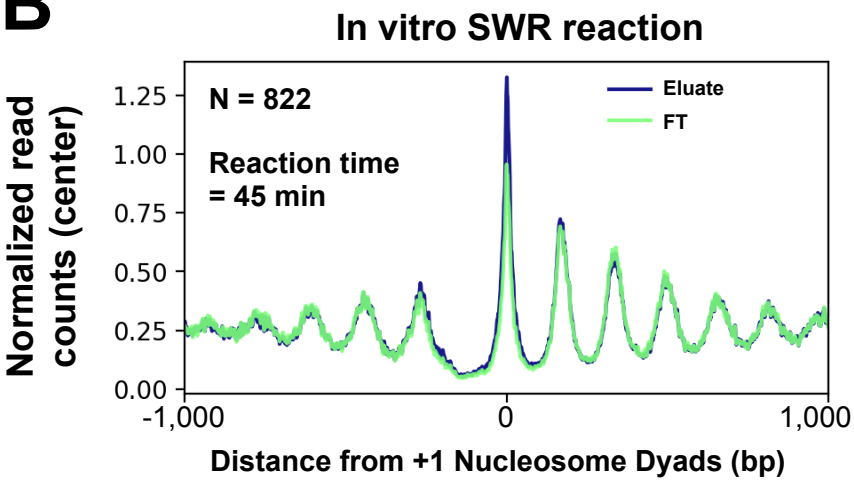

**C**

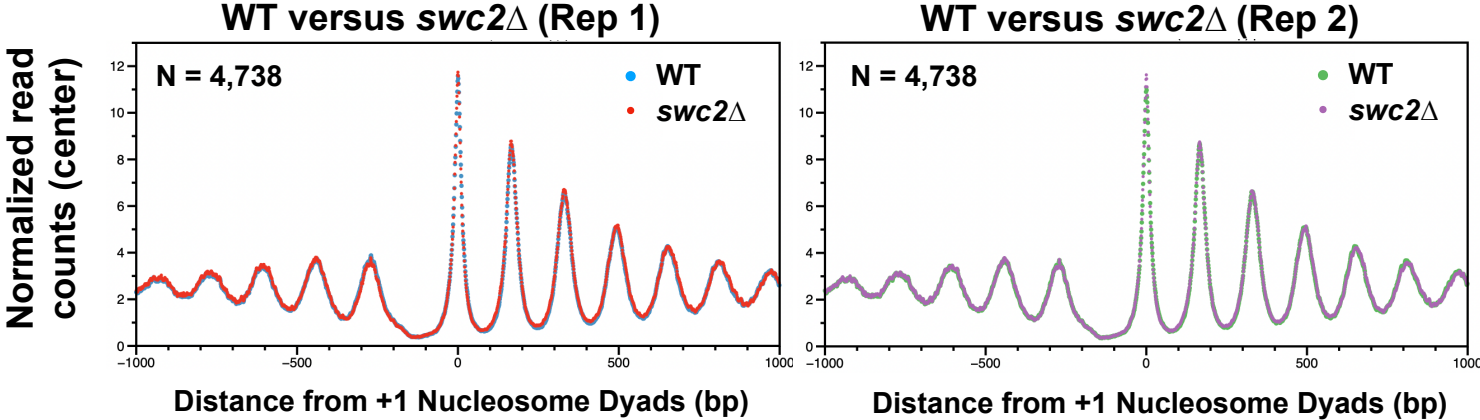

Supplement: S17 Fig — (A) MNase-seq analysis of WT and htz1Δswr1Δ mutant. Sequencing read counts were centered at the dyads of 4,738 annotated +1 nucleosomes. (B) Sequencing reads from the eluate and FT fractions of the in vitro SWR-mediated reactions (after Streptavidin pulldown) were mapped to 822 SWR-preferred +1 nucleosome sites identified through k-means analysis in Fig 4. (C) Input sequencing reads from WT and swc2Δ used in the H2A.Z IP reactions were centered at the dyads of +1 nucleosomes. Rep: biological replicate. The plot data for S17 Fig are available in S23 Data. (PDF) [file pbio.3003059.s017.pdf]
