# Supplementary material for: H2A.Z deposition by the SWR complex is stimulated by polyadenine DNA sequences in nucleosomes
Source: PLoS Biol. 2025 May 12;23(5):e3003059. doi: 10.1371/journal.pbio.3003059 (PMC12068740; doi:10.1371/journal.pbio.3003059)
Supplement: S1 Table — (PDF) [file pbio.3003059.s018.pdf]

S1 Table. Yeast strains

**Name**	**Genotype**	**Source**
yEL378	MAT**a** *his3Δ200, trp1Δ63, lys2-128δ, ura3-52, and leu2Δ1 (hta1-htb1)Δ::LEU2 (hta2-htb2)Δ::TRP1 htz1::HTZ1-2xFL <CEN ARS 2xV5-HTA1-HTB1 HIS3>*	This study
yEL379	MAT**a** *his3Δ200, trp1Δ63, lys2-128δ, ura3-52, and leu2Δ1 (hta1-htb1)Δ::LEU2 (hta2-htb2)Δ::TRP1 htz1::htz1(T46C)-2xFL <CEN ARS 2xV5-hta1(N39C)-HTB1 HIS3>*	This study
yEL427	*W1588-4C swr1::SWR1-3FLAG-p RVB1-MBP htz1Δ::KanMX6*	Sun, L. et al. 2020
yEL575	MAT**a** *his3Δ200, trp1Δ63, lys2-128δ, ura3-52, and leu2Δ1 (hta1-htb1)Δ::LEU2 (hta2-htb2)Δ::TRP1 htz1::htz1(T46C)-2xFL swc2Δ::kanMX6 <CEN ARS 2xV5-hta1(N39C)-HTB1 HIS3>*	This study
yEL704	MAT**a** *leu2Δ1 his3Δ200 ura3-52 trp1Δ63 lys2-128δ (hht1-hhf1)Δ::LEU2 (hht2-hhf2)Δ::HIS3 Ty912Δ35-lacZ::his4 <CEN ARS HHT2-2xV5-HHF2 TRP>*	This study
yEL786	MAT**a** *his3Δ200, trp1Δ63, lys2-128δ, ura3-52, and leu2Δ1 (hta1-htb1)Δ::LEU2 (hta2-htb2)Δ::TRP1 htz1::HTZ1-2xFL swc2Δ::kanMX6 <CEN ARS 2xV5-HTA1-HTB1 HIS3>*	This study
yEL905	MAT**a** *leu2Δ1 his3Δ200 ura3-52 trp1Δ63 lys2-128δ (hht1-hhf1)Δ::LEU2 (hht2-hhf2)Δ::HIS3 Ty912Δ35-lacZ::his4 htz1Δ::hphMX4 swr1Δ::p-kanMX-p <CEN ARS HHT2-2xV5-HHF2 TRP>*	This study
yEL1041	MAT**a** *his3Δ200, trp1Δ63, lys2-128δ, ura3-52, and leu2Δ1 (hta1-htb1)Δ::LEU2 (hta2-htb2)Δ::TRP1 htz1::htz1(T47C)-2xFL swr1Δ::kanMX6 <CEN ARS 2xV5-hta1(N39C)-HTB1 HIS3>*	This study
YYY67	MAT**a** *leu2Δ1 his3Δ200 ura3-52 trp1Δ63 lys2-128δ (hht1-hhf1)Δ::LEU2 (hht2-hhf2)Δ::HIS3 Ty912Δ35-lacZ::his4 <CEN ARS HHT1-HHF1 URA3>*	Yu, Y et al. 2011
