# Supplementary material for: H2A.Z deposition by the SWR complex is stimulated by polyadenine DNA sequences in nucleosomes
Source: PLoS Biol. 2025 May 12;23(5):e3003059. doi: 10.1371/journal.pbio.3003059 (PMC12068740; doi:10.1371/journal.pbio.3003059)
Supplement: S2 Table — (PDF) [file pbio.3003059.s019.pdf]

S2 Table. Plasmids

Name	Description	Source
pEL628	pRS414 *HHT2-2xV5-HHF2*	This study
pMS329	*CEN ARS HHT1-HHF2 URA3*	Yu, Y et al. 2011
pT22-6HisLyticase	pET22b 6xHis tagged gene fragment encoding amino acid 37-548 of glucan endo-1,3-beta-glucosidase from *Cellulosimicrobium cellulans* https://www.addgene.org/213504/	This study
