# Supplementary material for: H2A.Z deposition by the SWR complex is stimulated by polyadenine DNA sequences in nucleosomes
Source: PLoS Biol. 2025 May 12;23(5):e3003059. doi: 10.1371/journal.pbio.3003059 (PMC12068740; doi:10.1371/journal.pbio.3003059)
Supplement: S3 Table — (PDF) [file pbio.3003059.s020.pdf]

S3 Table. Primer sequences

Primers	Sequences	References
EL338 (reverse)	tacatgc ACA GGATGTATAT ATCTGAC	Sun, L. et al. 2020
EL873 (reverse)	tacatgc ACA GGATGTATAT ATCTGACACG TGCCTGGAGA CTAGGGAGTA ATCCCCTTGG/ideoxyU//ideoxyU/GTTAAAACGC	Sun, L. et al. 2020
EL1095 (forward)	ggccgcc CTG GAGAATCCCG GTGCCGAGGC CGCTCAATTG GTCGTAGCAA	This study
EL1223	/5AmMC6/TCTTCACACCGAGTTCATCCCTT	
EL1606 (forward)	/5AmMC6/ggccgcc CTG GAGAATC	This study
EL1607 (forward)	/5AmMC6/ggccgcc AAA AAAAAAAAAA G	This study
pT22(NdeI)Gluc-Gibs-F	aactttaagaaggagatatacatatgGTCCCCGCGACCATCCCGCTGACGATCACC	This study
pT22(XhoI)Gluc-Gibs-R	tcagtggtggtggtggtggtgctcgagGAGCGTCCAGCGCTGGGCC	This study
