# Supplementary material for: H2A.Z deposition by the SWR complex is stimulated by polyadenine DNA sequences in nucleosomes
Source: PLoS Biol. 2025 May 12;23(5):e3003059. doi: 10.1371/journal.pbio.3003059 (PMC12068740; doi:10.1371/journal.pbio.3003059)
Supplement: S4 Table — (PDF) [file pbio.3003059.s021.pdf]

S4 Table. Gene Block DNA sequences

Name	Sequences	References
gEL172 [Canonical Widom 601]	Tcttcacaccgagttcatcccttatgtgatggaccctatacgcggccgcc CTG GAGAATCCCG GTGCCGAGGC CGCTCAATTG GTCGTAGCAA GCTCTAGCAC CGCTTAAACG CACGTACGCG C TGTCCCCCGC GTTTTAACCG CCAAGGGGAT TACTCCCTAG TCTCCAGGCA CGTGTCAGAT ATATACATCC TGT gcatgta	This study
gEL173	Tcttcacaccgagttcatcccttatgtgatggaccctatacgcggccgcc **AAA AAAAAAAAAA** GTGCCGAGGC CGCTCAATTG GTCGTAGCAA GCTCTAGCAC CGCTTAAACG CACGTACGCG C TGTCCCCCGC GTTTTAACCG CCAAGGGGAT TACTCCCTAG TCTCCAGGCA CGTGTCAGAT ATATACATCC TGT gcatgta	This study
gEL174	Tcttcacaccgagttcatcccttatgtgatggaccctatacgcggccgcc CTG GAGAATCCCG **AAAAAAAAAA** CGCTCAATTG GTCGTAGCAA GCTCTAGCAC CGCTTAAACG CACGTACGCG C TGTCCCCCGC GTTTTAACCG CCAAGGGGAT TACTCCCTAG TCTCCAGGCA CGTGTCAGAT ATATACATCC TGT gcatgta	This study
gEL175	Tcttcacaccgagttcatcccttatgtgatggaccctatacgcggccgcc CTG GAGAATCCCG GTGCCGAGGC **AAAAAAAAAA** GTCGTAGCAA GCTCTAGCAC CGCTTAAACG CACGTACGCG C TGTCCCCCGC GTTTTAACCG CCAAGGGGAT TACTCCCTAG TCTCCAGGCA CGTGTCAGAT ATATACATCC TGT gcatgta	This study
gEL176	Tcttcacaccgagttcatcccttatgtgatggaccctatacgcggccgcc CTG GAGAATCCCG GTGCCGAGGC CGCTCAATTG **AAAAAAAAAA** GCTCTAGCAC CGCTTAAACG CACGTACGCG C TGTCCCCCGC GTTTTAACCG CCAAGGGGAT TACTCCCTAG TCTCCAGGCA CGTGTCAGAT ATATACATCC TGT gcatgta	This study
gEL177	Tcttcacaccgagttcatcccttatgtgatggaccctatacgcggccgcc CTG GAGAATCCCG GTGCCGAGGC CGCTCAATTG GTCGTAGCAA **AAAAAAAAAA** CGCTTAAACG CACGTACGCG C TGTCCCCCGC GTTTTAACCG CCAAGGGGAT TACTCCCTAG TCTCCAGGCA CGTGTCAGAT ATATACATCC TGT gcatgta	This study
C. cellulans glucanase (aa 37-548)	GTCCCCGCGACCATCCCGCTGACGATCACCAACGACTCGGGCAGGGGGCCGATCTACCTGTACGTCC<br>TCGGCGAGCGCGACGGCGTCGCCGGCTGGGCGGACGCGGGCGGCACGTTCCACCCGTGGCCCGGCGG<br>GGTCGGGCCCGTGCCCGTCCCGGCACCCGACGCGTCCATCGCGGGCCCCGGCCCCGGCCAGTCCGTG<br>ACGATCCGACTCCCGAAGCTGTCCGGGCGCGTCTACTACTCGTACGGCCAGAAGATGACGTTCCAGA<br>TCGTGCTCGACGGGCGGCTCGTCCAGCCCGCCGTCCAGAACGACTCCGACCCCAACCGGAACATCCT<br>CTTCAACTGGACCGAGTACACGCTCAACGACGGCGGCCTGTGGATCAACAGCACGCAGGTGGACCAC<br>TGGTCCGCGCCGTACCAGGTGGGCGTCCAGCGCGCCGACGGGCAGGTCCTCAGCACGGGCATGCTCA<br>AGCCGAACGGCTACGAGGCGTTCTACACGGCCCTCGAGGGCGCGGGGTGGGGCGGGCTCGTGCAGCG<br>CGCGCCCGACGGGAGCCGCCTGCGCGCGCTCAACCCGTCGCACGGGATCGACGTCGGGAAGATCTCG<br>TCGGCCTCGATCGACTCCTACGTCACCGAGGTGTGGAACTCGTACCGCACGCGCGACATGGTCGTCA<br>CGCCGTTCTCCCACGAGCCCGGCACGCAGTTCCGCGGCCGGGTCGACGGCGACTGGTTCCGCTTCAG<br>GAGCGGGTCCGGGCAGGAGGTCGCCGCGTTCAAGAAGCCCGACGCGTCGAGCGTGTACGGGTGCCAC<br>AAGGACCTCCAGGCGCCCAACGACCACGTCGTCGGGCCGATCGCCCGCACCCTGTGCGCCGCGCTCG<br>TCCGCACCACGGCGCTGACCAACCCGAACCAGCCGGACGCGAACAGCGCCGGCTTCTACCAGGACGC<br>GCGCACCAACGTCTACGCGAAGCTCGCGCACCAGCAGATGGCGAACGGCAAGGCGTACGCGTTCGCG<br>TTCGACGACGTCGGCGCGCACGAGTCGCTCGTCCACGACGGCAACCCCCAGGCCGCGTACATCAAGC<br>TCGACCCGTTCACCGGCACGGCCACGCCCCTCGGGAACGGCGGCAGCACCGAGCAGCCGGGCACCCC<br>CGGCGGTCTGCCCGCCGGGACGGGCGCGCTCCGCATCGGGAGCACGCTGTGCCTCGACGTCCCGTGG<br>GCCGACCCGACCGACACCAACCAGGTCCAGCTCGCCACCTGCAGCGGCAACGCGGCGCAGCAGTGGA<br>CGCGCGGCACCGACGGGACGGTGCGCGCCCTCGGCAAGTGTCTCGACGTCGCGCGCAGCGGGACGGC<br>CGACGGCACCGCCGTGTGGATCTACACGTGCAACGGGACGGGCGCGCAGAAGTGGACGTACGACTCC<br>GCGACCAAGGCCCTGCGCAACCCGCAGTCCGGCAAGTGCCTCGACGCCCAGGGCGGCGCACCGCTGC<br>GTGACGGCCAGAAGGTCCAGCTCTGGACCTGCAACCAGACCGAGGCCCAGCGCTGGACGCT	This study
