# Supplementary material for: H2A.Z deposition by the SWR complex is stimulated by polyadenine DNA sequences in nucleosomes
Source: PLoS Biol. 2025 May 12;23(5):e3003059. doi: 10.1371/journal.pbio.3003059 (PMC12068740; doi:10.1371/journal.pbio.3003059)

Fig 1B

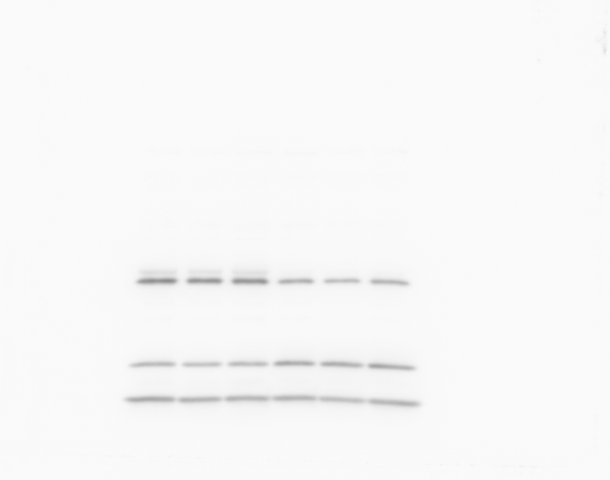

Fig 2B

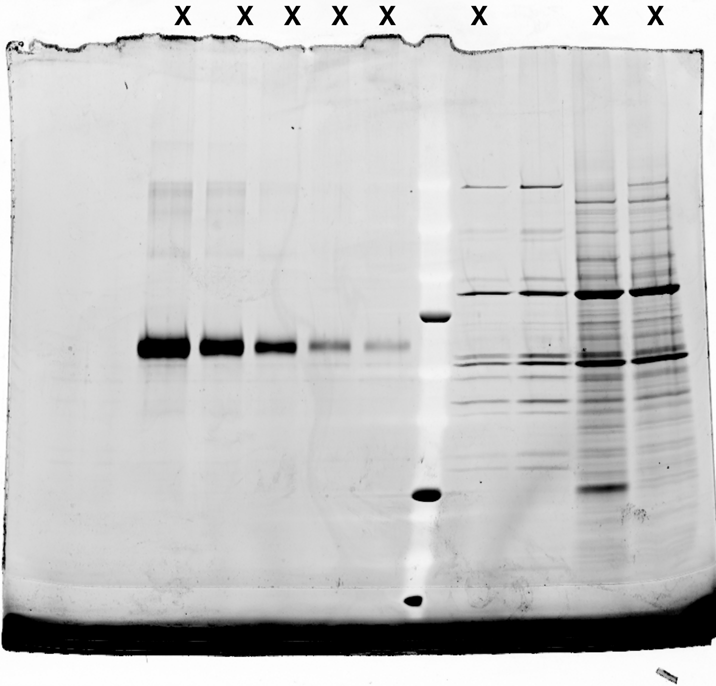

Fig 2C

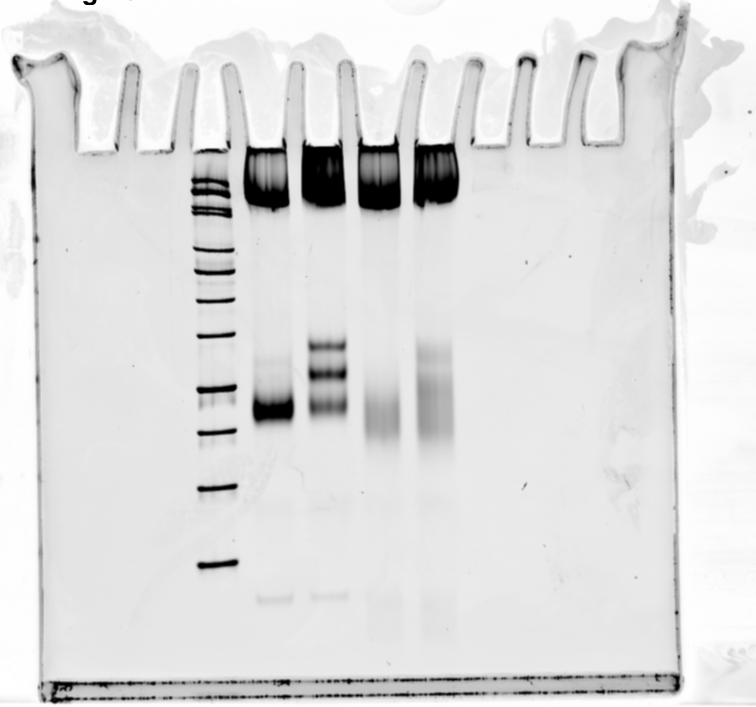

Fig 2E

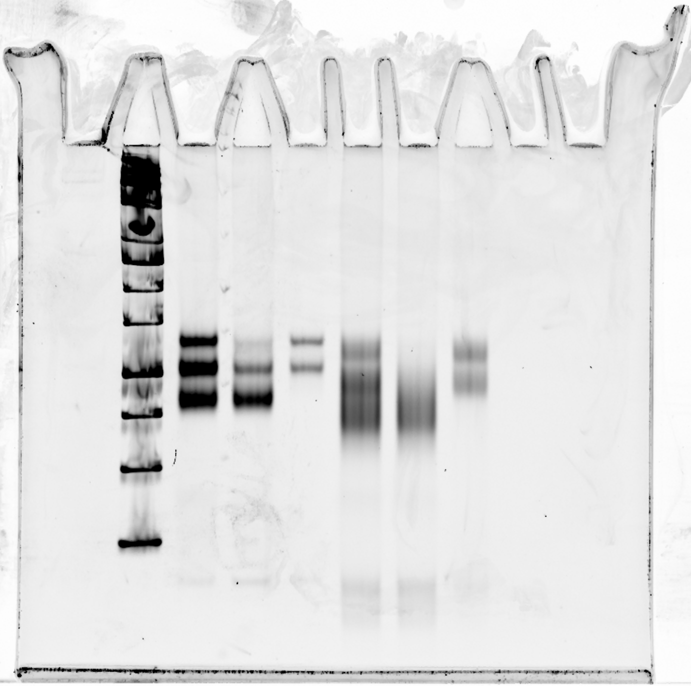

Fig 2F

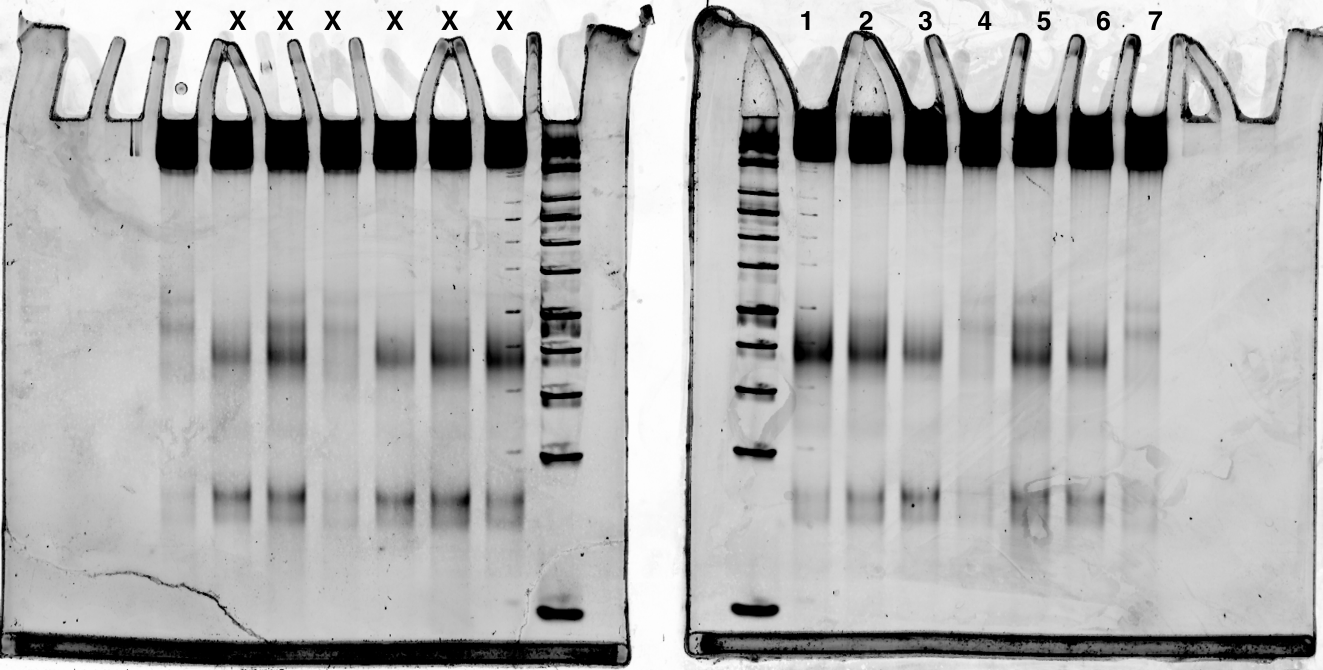

Fig 2F

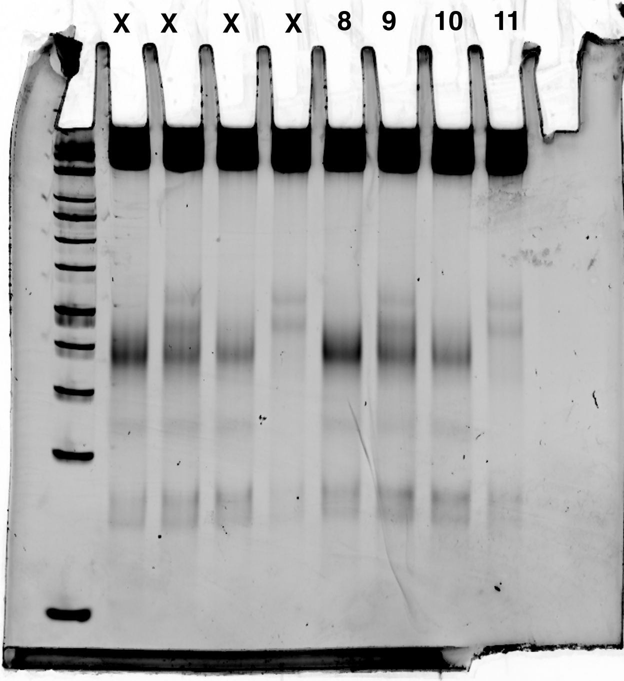

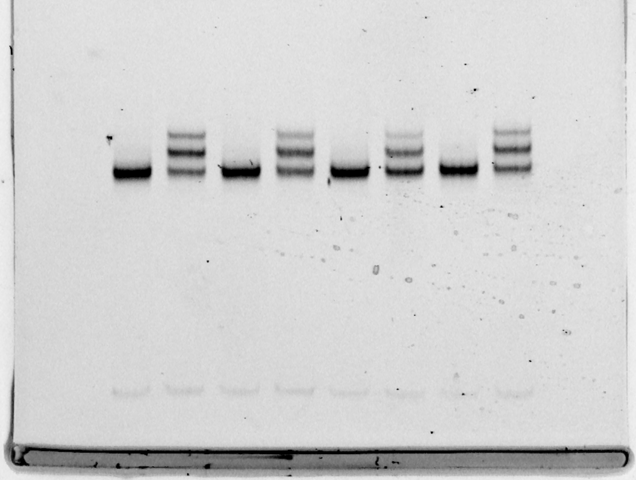

Fig 6D Alexa555

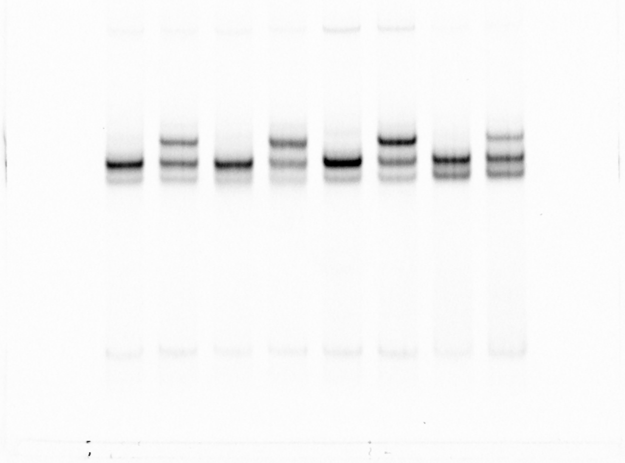

Fig 6D Alexa647

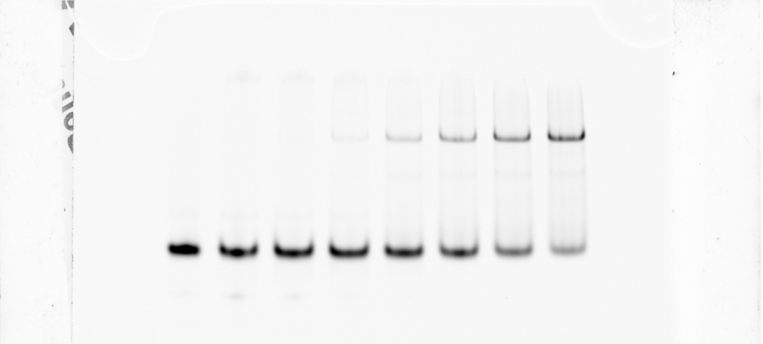

Fig 6E top

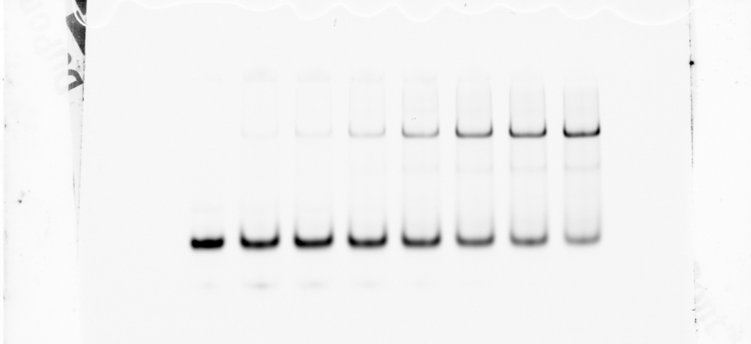

Fig 6E bottom

Fig 7D

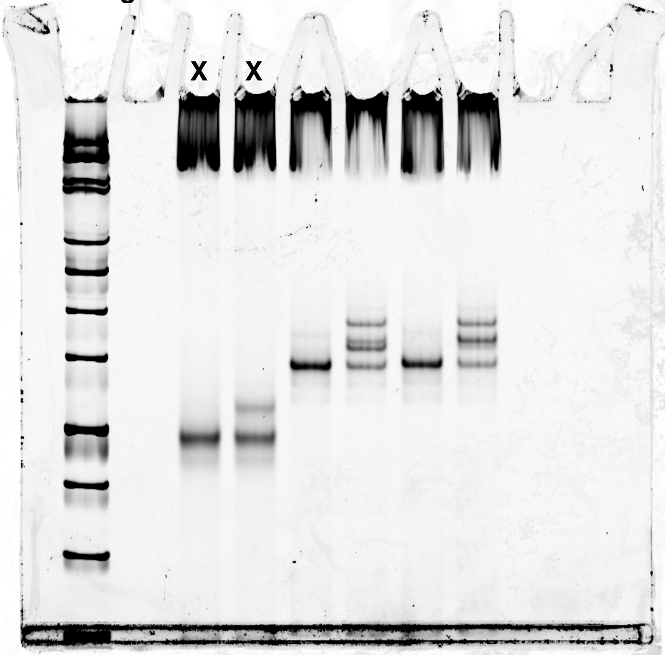

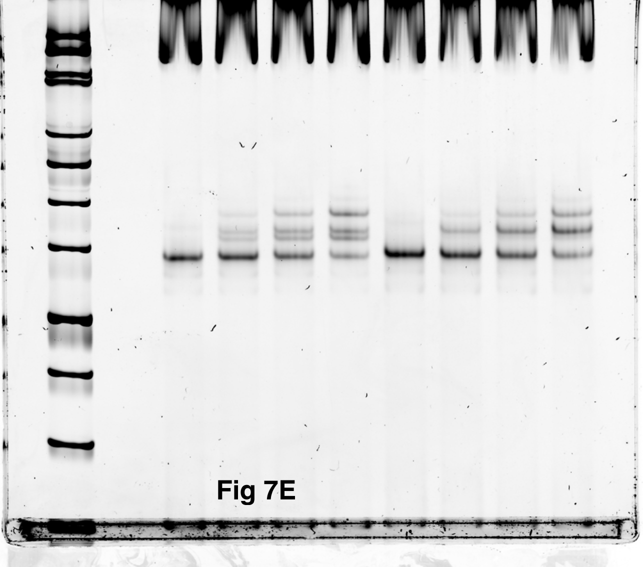
Fig 7E

S1 Fig A

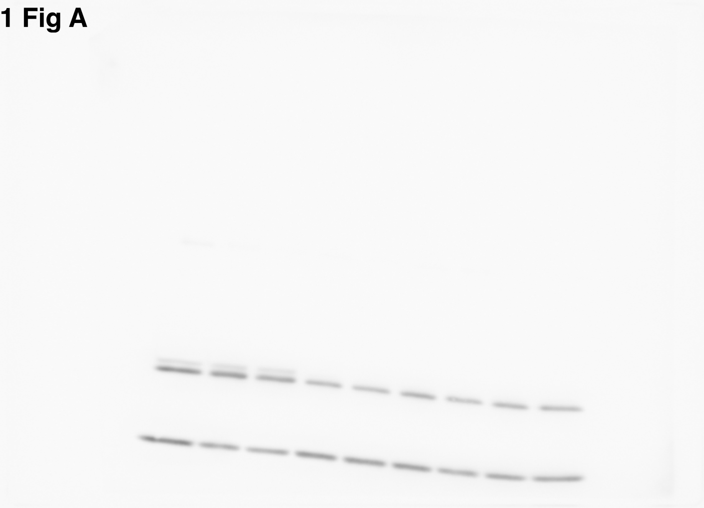

S1 Fig C
Rep 1

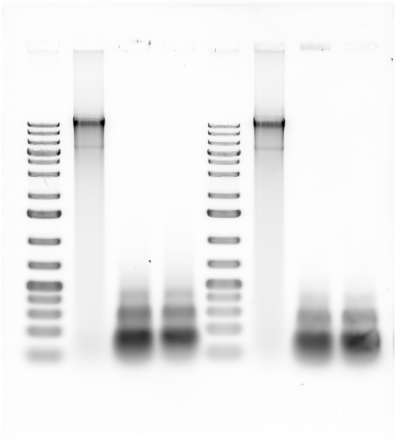

S1 Fig C
Rep 2

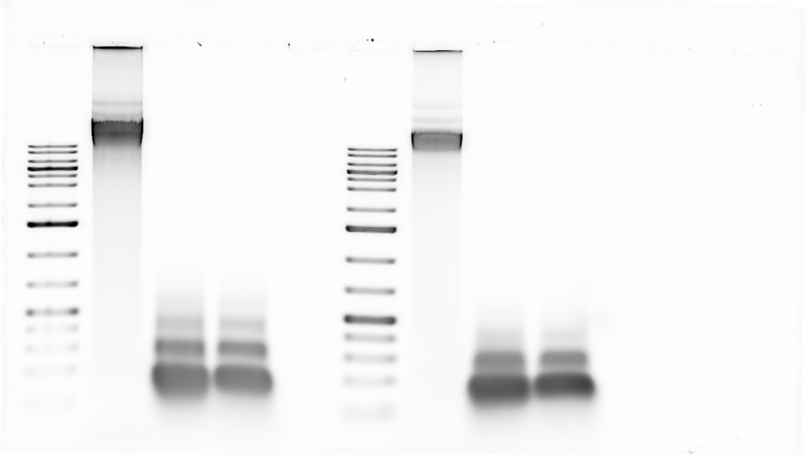

S1 Fig D
swc2Δ
rep1

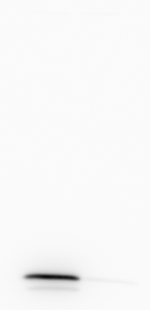

S1 Fig D
swc2Δ
rep2

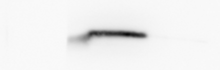

S1 Fig D
WT rep1

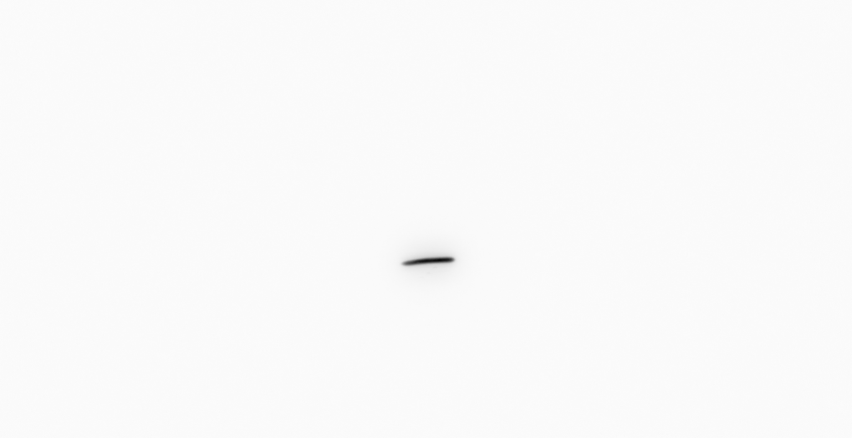

S1 Fig D
WT rep2

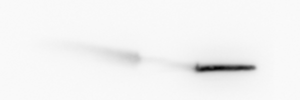

S4 Fig B

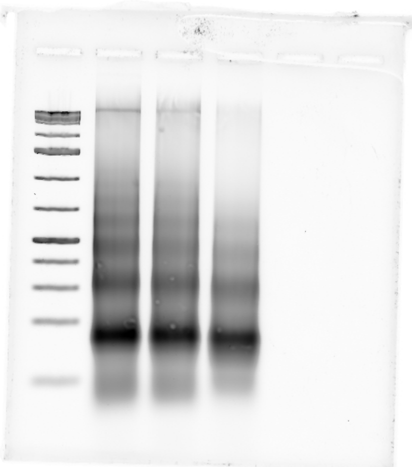

S4 Fig C, D

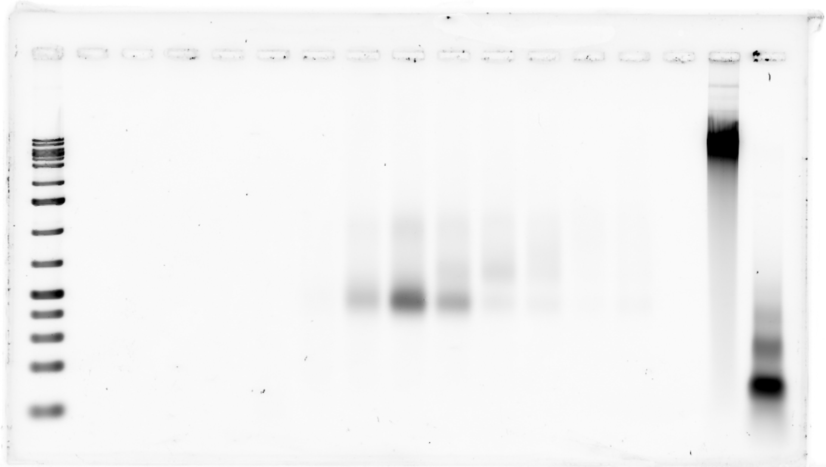

S5 Fig A

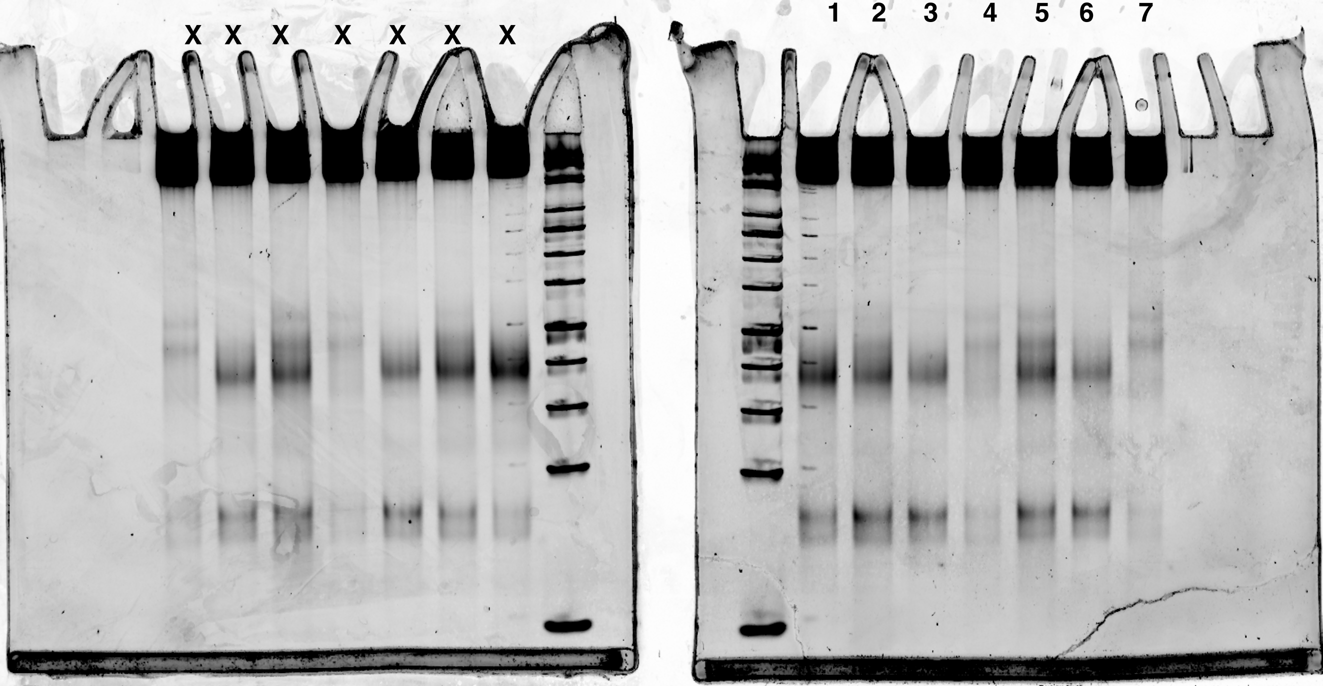

S5 Fig A

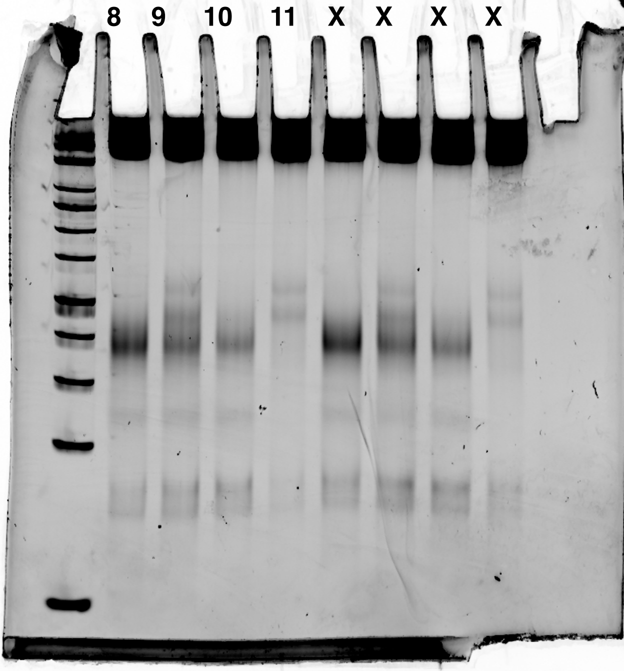

S5 Fig A

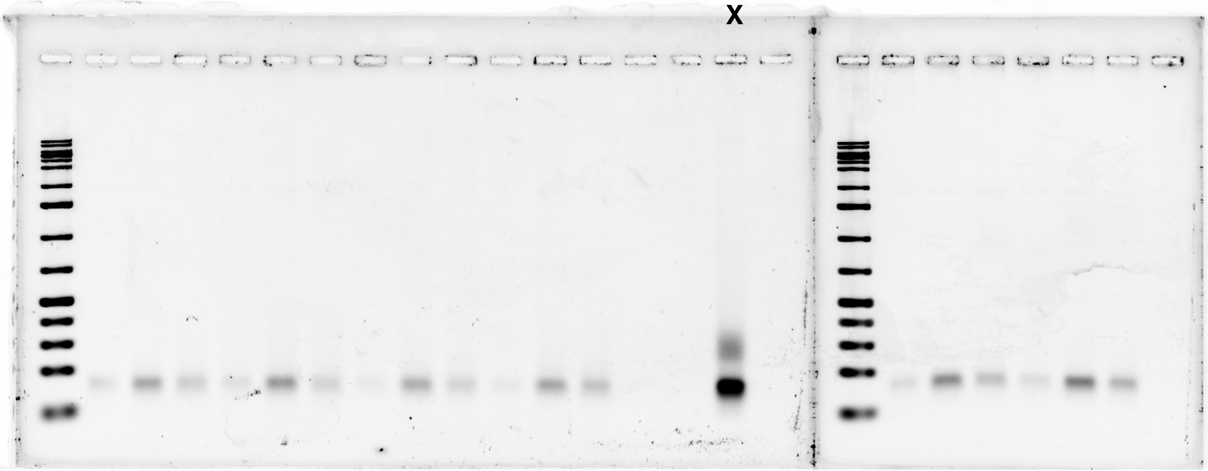

S12 Fig A
Alexa 555

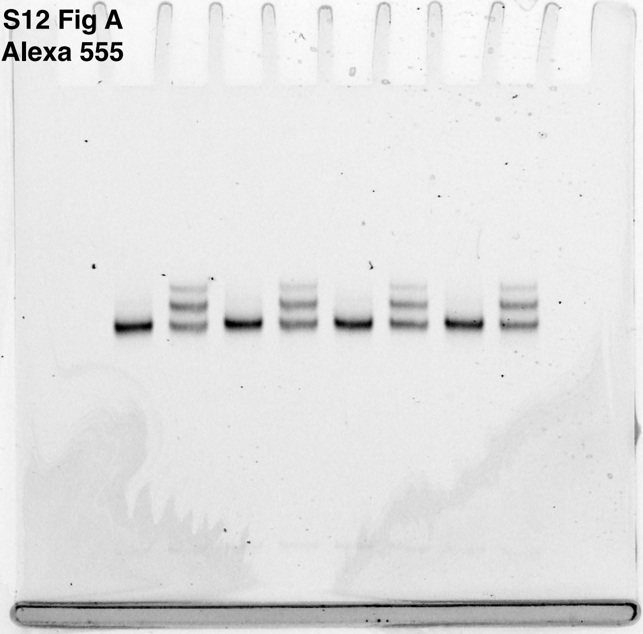

S12 Fig A
Alexa 647

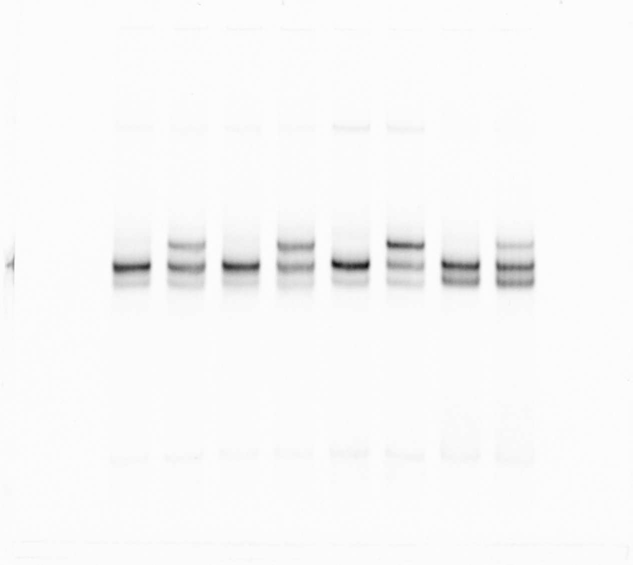

S12 Fig B
Alexa 555
rep 1

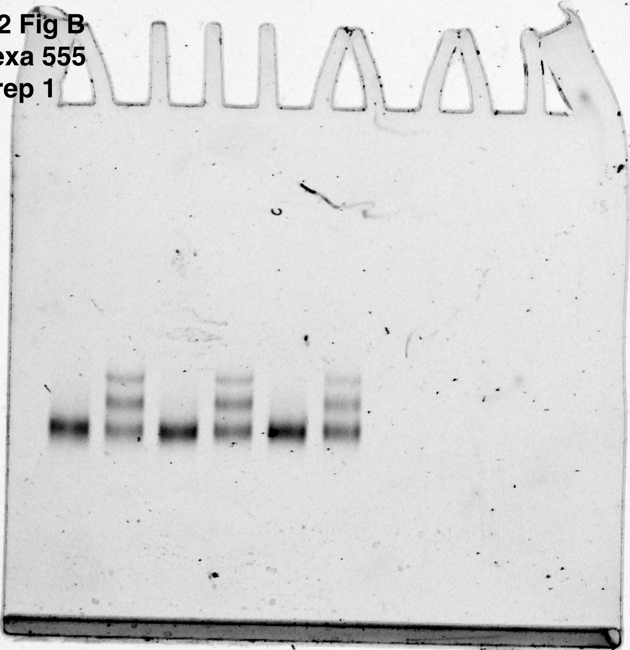

S12 Fig B
Alexa 555
rep 2

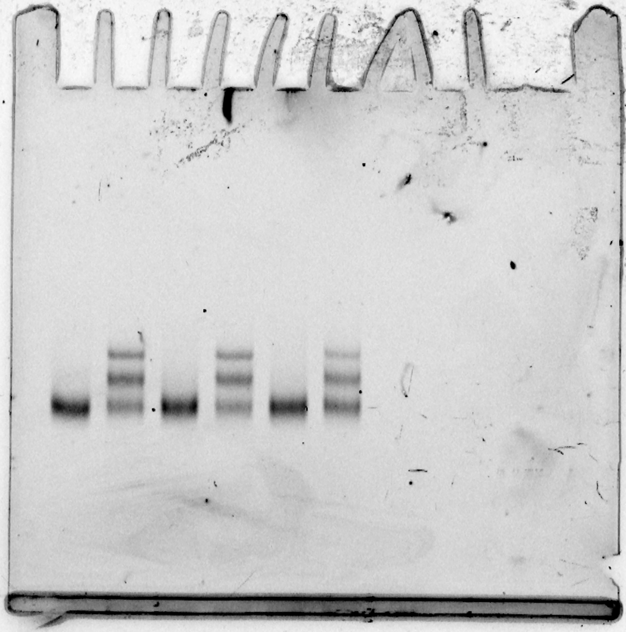

S12 Fig B
Alexa 647
rep 1

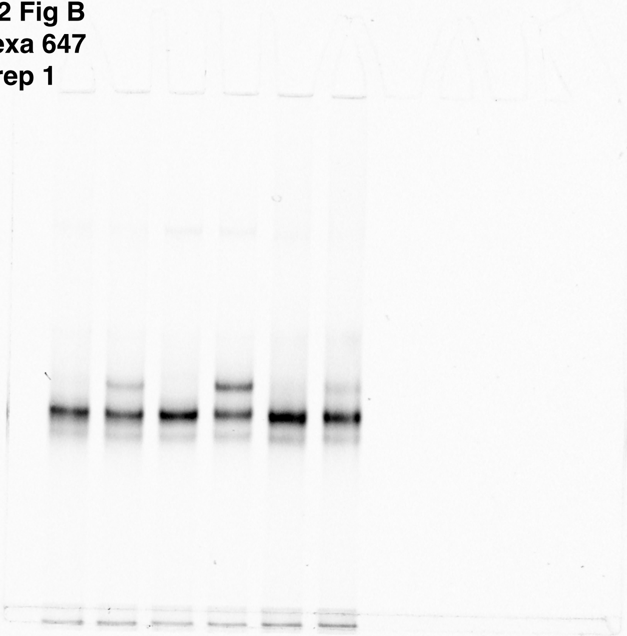

S12 Fig B
Alexa 647
rep 2

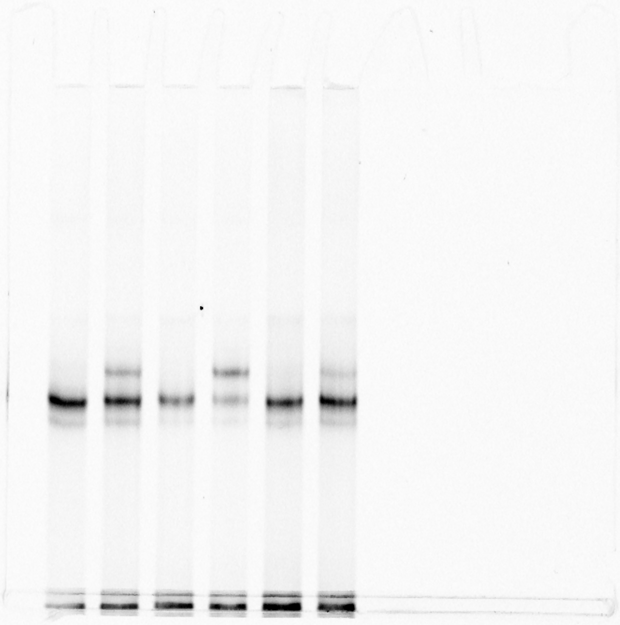

S12 Fig C
top

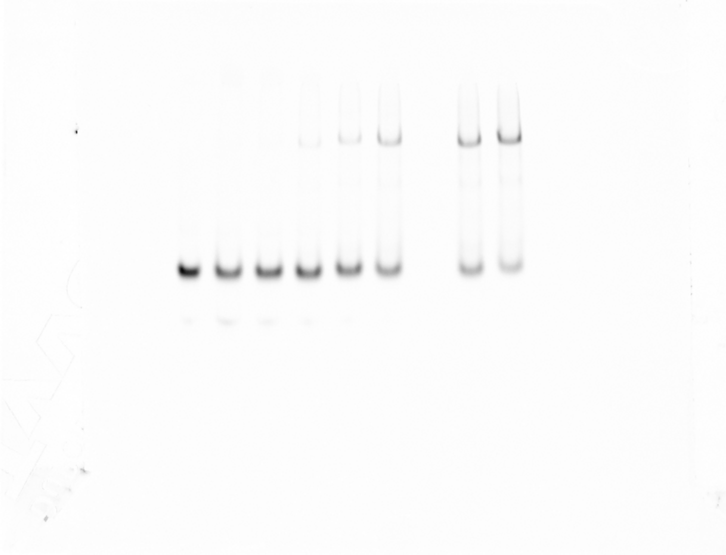

S12 Fig C
bottom

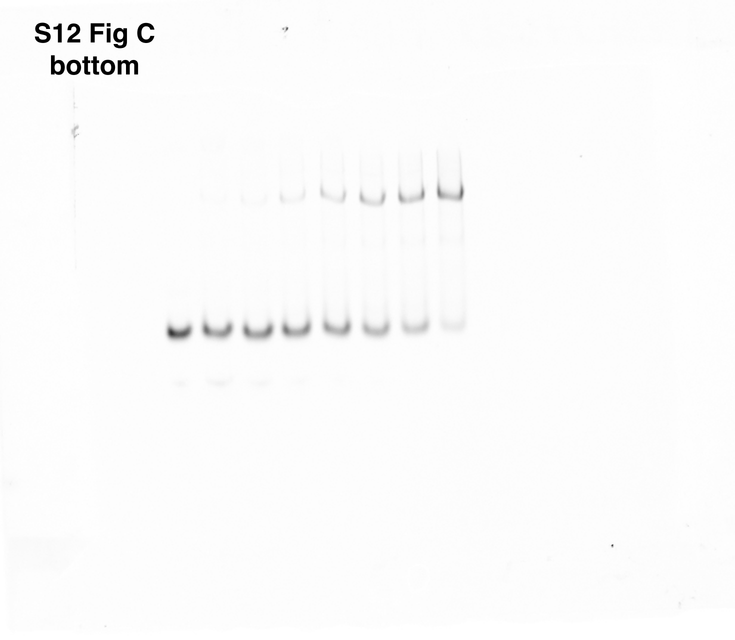

Supplement: S1 Raw images — (ZIP) [file pbio.3003059.s045.pdf]
