# Supplementary material for: H2A.Z deposition by the SWR complex is stimulated by polyadenine DNA sequences in nucleosomes
Source: PLoS Biol. 2025 May 12;23(5):e3003059. doi: 10.1371/journal.pbio.3003059 (PMC12068740; doi:10.1371/journal.pbio.3003059)
Supplement: S5 Fig — (A) Replicate 2 of the reaction time course as in Fig 2F. (B) The nucleosomal DNA from the indicated fractions were extracted and analyzed on a 1.3% agarose/0.5x TBE gel and stained with SYBR green. Sequencing libraries were prepared from the DNA in the FT and eluate fractions. (PDF) [file pbio.3003059.s005.pdf]

S5 Fig

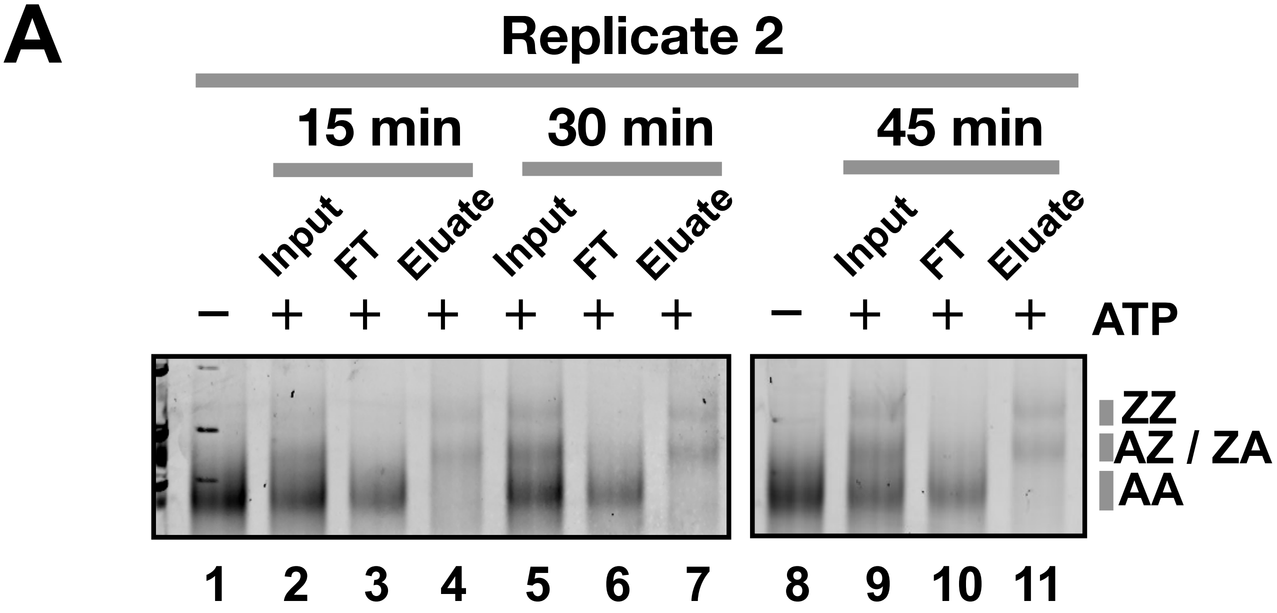
A
Replicate 2
15 min
30 min
45 min
Input
FT
Eluate
Input
FT
Eluate
Input
FT
Eluate
− + + + + + + − + + +
ATP
ZZ
AZ / ZA
AA
1 2 3 4 5 6 7 8 9 10 11

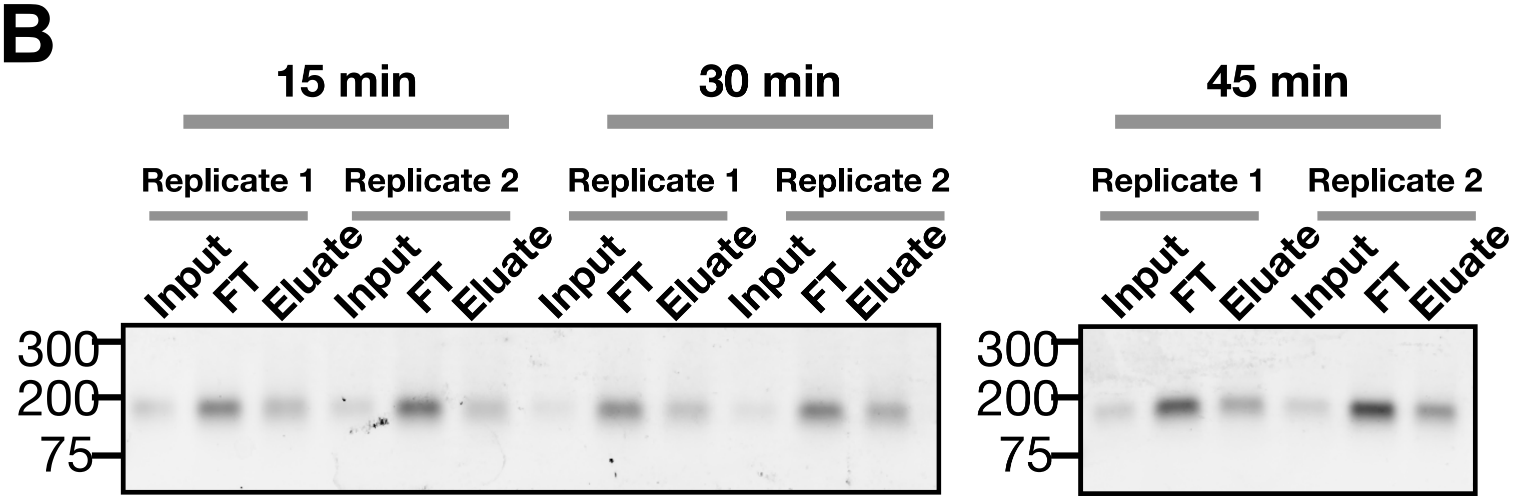
B
15 min
30 min
45 min
Replicate 1
Replicate 2
Replicate 1
Replicate 2
Replicate 1
Replicate 2
Input
FT
Eluate
300
200
75
