# Supplementary material for: H2A.Z deposition by the SWR complex is stimulated by polyadenine DNA sequences in nucleosomes
Source: PLoS Biol. 2025 May 12;23(5):e3003059. doi: 10.1371/journal.pbio.3003059 (PMC12068740; doi:10.1371/journal.pbio.3003059)
Supplement: Attachment — Submitted filename: Converso_response_to_reviewers_PLOS_Biol.pdf [file pbio.3003059.s046.pdf]

Reviewers' comments. (Our responses to the reviewers are highlighted in blue.)

Reviewer #1 (Remarks to the Author):

SWR is a conserved ATP-dependent chromatin remodeling enzyme that catalyzes the deposition of the H2A.Z histone variant in a novel dimer exchange reaction. In vivo, H2A.Z nucleosomes are highly enriched at promoter proximal nucleosomes. Both the biochemical mechanism of SWR action and its targeted to promoters is still unclear. Here, the authors ask several key questions: (1) How much H2A.Z incorporation within cells is SWR-dependent vs independent? And (2) What influence does DNA sequence have on the SWR-dependent incorporation of H2A.Z.

This is a very interesting piece work that uses several novel and sophisticated methodologies. Based on their data, the authors conclude that (1) over 50% of H2A.Z incorporation within cells occurs through SWR-independent pathways; and (2) SWR-dependent incorporation at in vivo sites can be explained in part by its preference to incorporate H2A.Z into nucleosomes that are enriched for A-T dinucleotides within the nucleosomal DNA that wraps on to H2A-H2B dimer that it targeted for replacement. This work has the potential to be highly impactful, but the data have several weaknesses that need to be addressed as detailed below.

1. The first part of paper presents two sets of studies to define SWR-dependent and independent deposition/chromatin incorporation of H2A.Z. First, they perform a sophisticated protein-protein crosslinking study which suggested the surprising conclusion that formation of over 50% of H2A.Z nucleosomes does not require SWR complex. They then perform an H2A.Z ChIP-seq in WT vs a swc2 deletion strain to determine where SWR-dependent and SWR-independent nucleosomes are located in the genome. However, there was no discussion or annotation of SWR-independent sites in the ChIP-seq study. From the few genome browser views shown, it would seem that the SWR-independent sites are randomly distributed? This should be discussed.

In the revised manuscript (lines 160-179), we have now discussed SWR-independent sites revealed by *swc2*Δ. The reviewer has correctly pointed out that SWR-independent sites are randomly distributed. This observation is consistent with our main point that SWR is responsible for virtually all distinct H2A.Z peaks in the yeast genome. We have now provided a new Figure S2 to further emphasize in a genome-wide manner that SWR-independent sites are randomly distributed. In the new figure, we show that when 59,044 non-repetitive nucleosomal sites were analyzed for H2A.Z enrichment, *swc2*Δ cells eliminated virtually all distinct H2A.Z peaks observed in WT cells.

Furthermore, this ChIP-seq analysis appears to lack sequence reads for input DNA, nor is there a ChIP-seq track for a FLAG IP from the untagged cell line. Given the small number of apparent reads from the swc2 deletion strain, this untagged control is essential for interpretation.

Contrary to Reviewer 1's comment, the Htz1-FLAG ChIP-seq read counts in the two *swc2Δ* replicates were not small (1.64×10^7 and 1.76×10^7 total aligned reads with lengths between 120-170 bp) in relation to the yeast genome (12 million bp). In fact, they were comparable to those in the WT replicates (1.87×10^7 and 1.78×10^7 aligned reads of the same length). Given that the IP efficiency of Htz1-FLAG in WT and *swc2Δ* was robust (see Figure S1D) and that FLAG peptides were used to elute the Htz1-FLAG containing nucleosomes from the FLAG beads, the bulk of the sequencing reads observed *swc2Δ* FLAG IP were unlikely originated from non-specific binding to the FLAG beads.

Therefore, the read coverage of *swc2Δ* appeared flat and low was due to two reasons. First, the DNA molecules associated with the residual chromatin-bound H2A.Z in *swc2Δ* are indeed randomly distributed with virtually no accumulation of H2A.Z at distinct locations, resulting in sequencing reads being spread evenly across the genome. Second, a scaling factor of 0.558, calculated based on our crosslinking data (Line 157), was applied to the *swc2Δ* H2A.Z ChIP-seq read coverage. This scaling factor accounts for the decrease in chromatin-bound H2A.Z due to loss of SWR activity, leading to an overall decrease in the amplitude of read coverage for *swc2Δ*.

2. The authors identify SWR-dependent peaks by subtracting the signal from the swc2 sample, but it is not clear from ~~this reviewer~~ (the authors) that this actually eliminated any peaks. Rather, this seemed to just remove the background signal.

The reviewer correctly pointed out that subtracting H2A.Z ChIP-seq signal of *swc2Δ* from that of WT effectively removes the background signal. This observation is crucial as it demonstrates that (1) the chromatin-bound H2A.Z deposited by SWR-independent pathways are randomly distributed and (2) SWR is responsible for depositing virtually all distinct H2A.Z sites across the yeast genome, including +1 nucleosomes, many non-+1 sites, and H2A.Z islands. We have emphasized this point in a revised paragraph (lines 172-179).

3. The authors use a swc2 deletion to define genomic locations where the SWR complex deposits H2A.Z. This assumes that the SWR complex that lacks swc2 is completely inactive. I do not think this has been shown definitively in vitro. What is the rationale for used a swc2 deletion rather than a swr1 deletion? At the least, the authors should rephrase the text to state that they are looking at Swc2-dependent sites of Z incorporation. There are many published ChIP-seq datasets from wildtype cells, swr1 deletion cells, and swc5 deletion cells. The authors should compare their data sets with these previously published ones. Is this new dataset unique in some way from these other datasets? In general, there is a disconnect between this initial in vivo section of the manuscript and the subsequent in vitro studies.

The rationale for using *swc2Δ* to identify genomic sites dependent on the SWR complex for H2A.Z deposition is grounded on previous biochemical studies that showed the SWR complex, devoid of the Swc2 subunit, exhibits no detectable H2A.Z insertion activity (DOI: 10.1038/nsmb1023). Nonetheless, we concur with Reviewer 1 on the

necessity of independently verifying the lack of activity of the SWR complex without Swc2. Therefore, we used our in vivo crosslinking assay (VivosX) to measure the amounts of chromatin-bound H2A.Z based on intra-nucleosomal crosslinking between two H2A.Z (which infers homotypic ZZ nucleosomes) or one H2A.Z and one H2A (which infers heterotypic AZ nucleosomes), and compared the nucleosomal H2A.Z levels in *swc2*Δ, *swr1*Δ, and WT.

In a new Figure S1A-B, we show that deletion of the *SWR1* gene, which encodes the ATPase motor of the SWR complex, exhibited similar levels of residual chromatin-bound H2A.Z compared to *swc2*Δ, suggesting that the SWR complex lacking the Swc2 subunit is as inactive as missing the ATPase core subunit. Therefore, the H2A.Z ChIP-seq data of *swc2*Δ is representative of SWR-independent H2A.Z distribution. We discuss this result in Lines 142-147 of the revised text.

4. Figures 2-4 present the most novel aspects of this paper. Based on these studies, the authors conclude that SWR-dependent H2A.Z nucleosomes generated in vivo are due in part to the sequence of DNA that wraps onto the H2A-H2B dimer interface. Remarkably, their novel assay appears to enrich for many +1 nucleosomes. However, it appears that these preferred nucleosomes are not really the bona fide +1 nucleosomes, but a nucleosome that is shifted 20-40bp into the NFR region. This places an A-T rich element (normally found in the nucleosome free NFR) in the SWR substrate nucleosomes. This seems odd, as these nucleosomes should not be present in the input native nucleosome sample. The authors suggest that these shifted +1 nucleosomes may have formed during nucleosome isolation (unlikely since these are non-preferred sequences for nucleosomes) or that they represent nucleosomes pre-RSC remodeling. However, the latter seems extremely unlikely as they would represent a tiny percentage of all +1 nucleosomes.

The positioning of +1 nucleosomes in a cell population is not absolute. For any given nucleosome, its native position varies slightly around a consensus position. Thus, when sequencing read counts are plotted in and around a consensus nucleosome position (e.g. the dyad of +1 nucleosomes), the sequencing reads distribute statistically around the consensus site in a pattern that approximates a normal distribution. For example, in the MNase-seq analysis shown on the right, we liberated nucleosomes from native chromatin by MNase digestion in a condition similar to that used to prepare our native nucleosome library (Figure R1A). The centers of sequencing reads distributed broadly around the consensus +1 position. To assess how much nucleosomes at +1 are slid >40bp upstream of the consensus +1

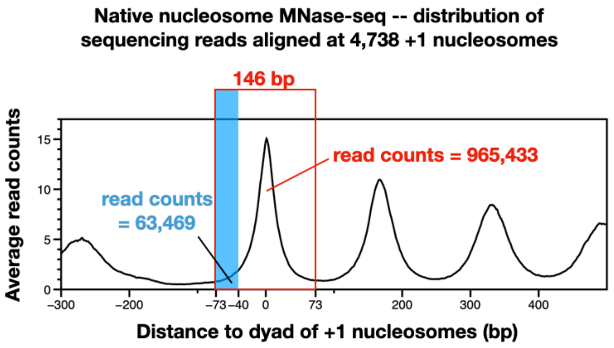

Figure R1: Average read counts were plotted along an 800-bp region aligned at the center of 4,738 consensus +1 nucleosomes. Each sequencing read is represented by a 3-bp regions in the middle of a paired-end read.

nucleosome center, we compared the read counts within a 33-bp region 40 bp upstream of the consensus +1 center and those within the 146-bp region of +1 nucleosomes. We found that ~6.5% of reads within the +1 nucleosomes are at least 40 bp upstream of the consensus +1 center. Therefore, the amount of +1 nucleosomes that are sled upstream towards the NFR are non-trivial.

I am concerned that a technical issue may have yielded the enrichment of these nucleosomes. Given the AT-rich sequences on one end, one possibility is that these sequences are actually short DNA linkers due to poor MNase cleavage (Mnase has lower activity on AT-rich sequences). Thus, these nucleosomes could reflect core particles with short linkers – substrates that SWR is known to have a 5x-higher affinity. It was not clear if the paired end sequencing analysis was performed in such a way to ensure that all particles where indeed only 145 bp in length. Perhaps sequencing of the original native nucleosome input could resolve this.

The Reviewer is correct to point out that SWR preferentially binds to nucleosomes with protruding linker DNA as opposed to nucleosome core particles. Therefore, we agree that our assay would bias for nucleosomes with slightly longer linker DNA. In the revised text, we have emphasized that our major conclusion that DNA sequences contribute to H2A.Z targeting by enhancing SWR activity is not inconsistent with or exclusive of the idea that NDR is a major recruitment mechanism of SWR (Lines 211-218, 367-370, and 423-429).

However contrary to the Reviewer's comment, MNase preferentially cut at the center on A/T dinucleotides over C/G dinucleotides (doi:10.1016/j.jmb.2012.01.043). Therefore, it is expected that AT-rich *linker* sequences would in fact be under-represented in nucleosomes with slightly longer linkers, not enriched. Importantly, our data show that the enrichment of AT-rich sequences in the SWR preferred substrates are localized deep within the nucleosomal region (up to SHL+/-1.5, Figure 6A-B). Furthermore, by inserting a 10-bp poly(dA:dT) track in the Widom positioning sequence at an internal site within the nucleosome, we showed that internal AT-rich sequences can indeed enhance the H2A.Z insertion activity of the SWR complex (Figure 7D). Altogether, these observations argue against the idea that preferred nucleosome observed in our in vitro assay was an artifact created by poor MNase cleavage in AT-rich sequences in linker DNA regions.

Previously, the authors previously published that SWR showed higher activity on nucleosomes that contained a higher G-C content near the ATPase binding site at SHL2 (Sun et al., 2020). Was this sequence motif enriched in their dataset? This needs to be discussed in order to reconcile their two studies.

We appreciate the Reviewer for pointing out this apparent contradiction of our earlier study and our initial explanation in the introduction was insufficient (Lines 86-93). Therefore, we have provided the following paragraphs in the discussion to explicitly reconcile the two studies:

In previous studies, we observed that SWR inserts Z-B dimers onto the two faces of a nucleosomal substrate reconstituted with the asymmetric Widom 601 sequence at different efficiencies at lower temperatures, such as 4 and 10˚C [28]. We identified a 16-bp region, spanning positions 24 to 39 bp relative to the nucleosomal dyad, that when swapped with the corresponding site on the opposite face of the nucleosome, is sufficient to cause the asymmetric H2A.Z insertion effect. The 16-bp sequence that promotes H2A.Z insertion at low temperatures (5'-TAGGG AGTAA TCCCC T-3') contains two short poly(dG:dC) tracks, unlike the less favored counterpart (5'-TCGTA GCAAG CTCTA G-3'), despite both having equivalent GC content. This observation leads to the speculation that SWR prefers poly(dG:dC) tracks. However, under our current experimental conditions at an ambient temperature of 23˚C, SWR did not prefer native nucleosomes with high dCdC and dGdG contents. The discrepancy could be attributed to the temperature at which the current SWR assays were performed. Alternatively, the preferred 16-bp region (overlapping the A-B/Z-B dimer binding site but not Pos 3) contains more nuanced motifs other than poly(dG:dC) tracks that are favored by SWR at low temperatures. Thus, the sequence motifs favored by SWR likely have multiple permutations that can be influenced by specific reactions conditions, with poly(dA:dT) tracks identified here representing a key element that could combine with other motifs to fine tune SWR-mediated H2A.Z insertion in vivo.

When investigating substrate preference of SWR with the native nucleosome library, SWR did not target many +1 nucleosomes that are typically enriched for H2A.Z, especially at the earliest (15 min) reaction time point (Figure S8A). This failure likely results from the disruption of long linkers by MNase digestion, which are presumably more important for SWR recruitment than sequence motifs at these sites. In fact, these +1 nucleosomes are generally associated with higher frequency of poly(dG:dC) tracks [28], which do not favor H2A.Z insertion under our reaction conditions (Figure 6), further reinforcing the idea that a subpopulation of endogenous H2A.Z sites do not rely on nucleosomal sequence motifs to facilitate H2A.Z insertion. Therefore, across different genomic locations, a complex interplay between nucleosomal linker DNA length, sequence motifs, and histone modifications orchestrates the site-specific deposition of H2A.Z in vivo.

The authors should also comment if the GC-rich side of the 601 is the same side where the A-T rich elements were inserted. If so, does this impact their effectiveness?

The poly(dA:dT) tracks were added to the 'AT-rich' side of the Widom 601 sequence. We have added a comment in the legend of Figure 7 to clarify this point (Line 984). The AT-rich side of the Widom sequence refers to the three extra TA dinucleotide anchors at SHL-0.5, -2.5 and SHL-3.5, which are located at the inward facing minor grooves of the nucleosomal DNA curvature and are known to be more accommodating to coil around the histone core than the C/G dinucleotides on the opposite side (doi:10.1093/nar/gks261). However, the interplay between the TA anchors and the poly(dA) tracks on the biophysical and enzymatic properties of nucleosomes are likely complicated and will require systematic mutagenesis study to fully understand their

combinatorial effects on SWR activity. Therefore, the reviewer's question is a good one but is beyond the scope of this study.

Figure 7. For the in vitro exchange assays with 601 nucleosomes harboring A-rich sequence elements, it appears that these reactions were performed under catalytic reaction conditions (20x excess nucleosomes). The simplest interpretation of the data, especially due to the impact on the internal control substrate, is that the A-rich insertions increase the nucleosome binding affinity for SWR, not the kcat (also true for the native nucleosome analyses). This should be tested directly, as it has a major impact on the resulting model. This could be done by nucleosome binding assays or by repeating the exchange reactions under single turnover conditions.

We agree with Reviewer 1 on the importance of understanding the mechanistic details of how the poly(dA:dT) sequence enhances H2A.Z insertion by the SWR complex. One possibility, as suggested by the reviewer, is that poly(dA) increases SWR's affinity for the nucleosome. However, even if poly(dA) does enhance affinity, it does not preclude the possibility that poly(dA) could also stimulate SWR's catalytic activity (k_{cat}). In the histone exchange reaction catalyzed by SWR, three substrates are involved: the canonical nucleosome, the Z/B dimer, and ATP. Poly(dA) may directly influence nucleosome binding, but it could also indirectly modulate A/B dimer ejection, Z/B dimer insertion, and the ATP hydrolysis rate. In the simplest scenario where poly(dA) only aids in nucleosome binding, questions remain as to whether poly(dA) facilitates the unraveling of nucleosomal DNA to provide a landing pad for SWR or induces structural distortions on the nucleosome surface for recognition. Therefore, the reviewer's points are well taken, but addressing these questions rigorously will require extensive further study beyond the scope of this paper.

Minor point:

Previously, the yeast enzyme that deposits H2A.Z has been called 'SWR1', 'SWR-C', and 'SWR1C'. In this paper, the authors introduce a new acronym – 'SWR'. It is not clear that this is any better. I agree with the authors that 'SWR1' is confusing with the gene name for the ATPase subunit, Swr1. In my opinion, SWR1C is preferable, as it does not require the use of special characters (hyphen), but SWR-C is certainly better than SWR or SWR1.

The 'SWR' acronym is in fact not new. It was used by Geeta Narlikar et al. in a review (doi:10.1146/annurev-biophys-051013-022819) as well as by my group in multiple publications to describe the SWR complex. Since the nomenclature was clear in distinguishing the complex from its ATPase subunit (Swr1), we respectfully decline to give up on a nomenclature that we have used consistently and to conform to a different one.

Reviewer #2 (Remarks to the Author):

The authors examine how SWR1 is targeted to genomic DNA and if there is an intrinsic DNA sequence basis similar to that observed for other ATP-dependent chromatin remodelers like RSC and CHD1. In the first figure the authors use a novel approach to determine the fraction of H2A.Z (Htz1) in yeast that is chromatin-bound by disulfide crosslinking of the two H2A histones inside of nucleosomes and attaching 2 FLAG tags to the C-terminus of Htz1. By Western blotting they can determine the amount of uncrosslinked Htz1 (not bound to chromatin) and the proportion of either 1 or 2 Htz1 per nucleosome. Their approach is very clever and reveals 12% is with 2 copies of H2A.Z per nucleosome, 57% with one copy of H2A.Z per nucleosomes and 31% not in nucleosomes. They use this data to better normalize ChIP-seq from wild type and swc2Δ strains. Using a swc2Δ strain they show SWR1 is only responsible for incorporating half of the single H2A.Z containing nucleosome genome wide. Do they authors have any suggestion as to the enzyme or mechanism for the incorporation of H2A.Z that is not SWR1 dependent at more than half of its other target nucleosomes?

The mechanisms contributing to the incorporation of H2A.Z independent of SWR remain an intriguing question. As this topic warrants careful investigation and is not the main focus of our paper, we prefer not to speculate at this time. We aim to explore this question in future studies.

What is the data about how defective is SWR1 in the absence of the Swc2 subunit and is it completely inactive?

As mentioned in our response to Reviewer 1, we chose to inactive the SWR complex by ablating *SWC2* based on biochemical experiments demonstrating that SWR requires the Swc2 subunit for activity (DOI: 10.1038/nsmb1023). To independently verify this result, we have now included data showing that deletion of the *SWR1* gene, which encodes the core ATPase of the SWR complex, decreases chromatin-bound H2A.Z to a similar extent as *swc2*Δ (Figure S1A-B). This suggests that in the absence of the Swc2 subunit, the SWR complex is as inactive as it is without its ATPase core motor, which is essential for its H2A.Z insertion activity. Therefore, the residual chromatin-bound H2A.Z observed in *swc2*Δ or *swr1*Δ are indeed deposited by SWR-independent mechanism. We appreciate the reviewer for motivating us to further confirm this important point in our paper.

A key finding from this data is that a significant number of the sites where H2A.Z is incorporated in a Swc2 dependent manner are not associated with histone acetylation or promoter regions. They suggest there might be a different targeting mechanism involved at these sites.

They use a novel approach for querying yeast chromatin to determine if there are specific DNA sequences that SWR1 prefers when targeting the exchange of H2A for H2A.Z. Their approach is different than that originally pioneered by Phillip Korber and colleagues using a library of yeast genomic DNA and reconstituting it into chromatin by salt dialysis with recombinant histone octamer. In their case, they use a yeast strain that

eliminates H2A.Z and Swr1 and then isolate native mononucleosomes after MNase digestion. There are a few concerns about this approach being an effective way to mimic yeast chromatin in its native state. Isolated nucleosomes with no linker DNA will be behave quite differently than native yeast chromatin in which there is not an abundance of free DNA ends and linker DNA is a common feature of chromatin. I am especially concerned because of the breadth of the literature showing the importance of linker DNA not only for the scanning and recruitment of SWR1 (PMID: 24034247, PMID: 35876491, PMID: 2403424), but also for its enzymatic activity (PMID: 35263135). By far the majority of the paper is focused on this one approach. Figures 2-4 alone are all about the setup of this type of experiments and the initial genomic data that is collected and the subsequent Figures 5-6 detail further analysis of this data. The efficacy and veracity of this approach is therefore the major part of this paper.

It is true that long linker DNA is important for the recruitment and binding the SWR complex to its nucleosomal substrate. When SWR is presented with in vitro nucleosomal substrates with variable linker DNA lengths, it preferentially inserts H2A.Z into those associated with longer linker DNA. However, SWR is fully capable of inserting H2A.Z into nucleosomes with very short or even no linker DNA, as demonstrated by us and others (see PMID 31914392, Fig 2F-H and PMID 24034247, Fig S2B). The ability of SWR to insert H2A.Z into nucleosomes with minimal or no linker DNA is important for this study, as it allows us to isolate the nucleosomal DNA sequence effect from the linker DNA effect on SWR's H2A.Z insertion activity, where the latter is more dominant. We have now revised the text in Lines 211-218, 367-370, and 419-429 to clarify this point.

According to the ChIP-seq data there appears to be roughly 10,000 nucleosomal positions where SWR1 accounts for the incorporation of H2A.Z and is considerably more than the number of sites observed with the in vitro assay to be enriched for H2A.Z (564-781, Figure 4C). This type of discrepancy raises concern about the sensitivity of the in vitro approach, which is further compounded by only 32%-44% of this small number of sites corresponding to those found to be enriched for H2A.Z in vivo.

It is well established that long linker DNA and histone modifications, such as H3 and H4 tail acetylation, are important for the recruitment of the SWR complex, and we do not dispute these previous findings. Rather, the main point of this study is that nucleosomal DNA sequences can modulate the remodeling activity of the SWR complex and affect H2A.Z deposition. Therefore, in cells, the site-specific deposition of H2A.Z in vivo is influenced by at least three factors: linker DNA, histone modifications, and nucleosomal DNA sequence. In our native nucleosome library, the removal of long linker DNA by MNase eliminates one component of SWR recruitment, thereby revealing the effect of DNA sequences. Thus, the relatively small number of sites identified by our in vitro approach, which represent in vivo sites that rely heavily on the DNA sequence effects, is not unexpected. This finding demonstrates how nature has evolved to use a combination of factors to achieve site-specific H2A.Z deposition.

Also, the comparison of these versus random sites isn't very encouraging because of factor of ~2 difference (18% versus 32-44%) doesn't suggest a great deal of selectivity. The next concern is seen by the in vitro assay showing a strong artifactual preference for the 3' end of genes not observed in vivo. If there is this type of inaccurate results from the in vitro data, how do you know you can trust the data at the other sites as being a correct reflection of SWR1's targeting and activity and isn't this an important litmus test of the veracity of the in vitro approach?

The goal of our in vitro assay utilizing native nucleosome substrates is not to completely recapitulate the in vivo distribution of H2A.Z but to reveal any novel factors contributing to the site-specific insertion of H2A.Z. Our assay identified 1,777 nucleosomal positions preferred by SWR. Among those, 564 sites (32%) were enriched for H2A.Z endogenously at the 15-min time point. The total number of nucleosomes considered in our assay was 59,044 (covering non-repetitive regions of the yeast genome) and 10,662 of those (18%) were considered endogenously enriched for H2A.Z.

To determine if our in vitro assay is *better* at identifying endogenous H2A.Z sites (alternative hypothesis) than randomly picking nucleosomes (null hypothesis), we performed a one-tailed test. The success rate in identifying H2A.Z nucleosomes in our assay is 32% ($\hat{p}$) relative to 18% (p) in the population. Given our sample size (1,777), the Standard Error (SE) is 0.0091. The Z-score, calculated as $z = (\hat{p} - p) / SE$, for our in vitro assay performance is therefore $[(0.32 - 0.18) / 0.0091] \approx 15$. This results in a p-value of virtually zero, indicating that our in vitro assay (for the 15 min time point) is significantly better at identifying endogenous H2A.Z sites than random selection. Our 30-min and 45-min time points, with identification rates ($\hat{p}$) of 39% and 44%, respectively, are therefore even more significant at identifying H2A.Z sites. This analysis is now included in Lines 567-576.

The fact that our in vitro assay is making false positive errors (i.e., identifying H2A.Z at the 3' end of genes) does not negate the power of our assay. Instead, it reveals an unknown mechanism at the 3' end that prevents H2A.Z deposition. Investigating how this occurs is important and should be a subject of a future study.

Also what do you think it means when you observe for your in vitro assays that SWR1 strongly prefers to position H2A.Z off-center from the in vivo preferred sites?

As mentioned in our response to Reviewer 1, Comment 4, the position of nucleosomes in a cell population is not absolute but varies slightly around a consensus position. Our analysis indicates that 6.5% of +1 nucleosomes were shifted upstream of the +1 consensus site by 40 bp within a 33 bp region. The reason why SWR exhibits a preference for shifted nucleosomal substrates is unclear. We speculate that the shifted nucleosome has a higher proportion of poly(dA:dT) tracks, leading to higher SWR activities.

The effects polyA or poly T tracts from SHL-7.5 to -2.5 on SWR1 incorporation of H2A.Z are interesting, especially given their location relative to where H2A.Z is incorporated.

The follow up experiments with 601 nucleosomes and incorporating poly A tracts of 10 bp are extremely helpful and are nice in validating the other in vitro data. However, I didn't find any description either in the figure legend or methods as to whether these 601 nucleosomes had any linker DNA (length?) or if not. If these are just core 601 nucleosomes without any linker DNA then I would ask why were these experiments done in this manner? Given the role of linker DNA enhancing the activity of SWR1 if not done with 601 nucleosomes with sufficient length of linker DNA then I would ask what exactly is being assayed? It might be possible that at more optimal conditions with sufficient linker DNA present that the inhibitory effects of poly A tract may become significantly less.

All nucleosomal substrates used in Figure 7 and S13 were flanked by 7-bp linkers DNA on both sides. This design intentionally excludes long linker DNA to isolate and reveal the contribution of the sequence effect on the H2A.Z deposition activity of SWR. We have now added a sentence in the legend of Figure 7 to highlight this point (Lines 986-988). We do not dispute the importance of long linker DNA in SWR recruitment (see our explanation above); rather, the goal of this study is to uncover a previously unappreciated role of DNA sequence in SWR-mediated H2A.Z deposition.

The title indicates this paper is about targeting related to SWR1 and I wonder if targeting is really the focus of the data presented. It seems most of the data is about how DNA sequence, general poly A or poly T tracts, impact the enzymatic activity of SWR1 rather than how it is targeted. A key aspect missing in this paper that is critical is the potential interplay between these DNAs that inhibit SWR1 and linker DNA, especially if one wants to focus on targeting.

The point is well taken. We agree that the poly(dA:dT) sequences impact the enzymatic activity of SWR but not how the remodeler is targeted. However, by modulating the activity of SWR, this in turn impacts the targeting of H2A.Z. Therefore, we have revised the title of the paper to reflect these points. We appreciate the Reviewer's suggestion, which has significantly improved the paper title.

New title: *"Impact of Nucleosomal DNA sequences on SWR Remodeling Activity and H2A.Z targeting."*

Minor points:
(1) In Figure S7A it is vital to be more quantitative than merely stating there are more +1 nucleosomes inserted with H2A.Z at the later time points. You need to report the actual number and percentage seen in this heat map.

Agreed. Out of the 1,777 top 3% SWR-preferred nucleosome, 217 (12%) are associated with the +1 nucleosomal position at the 15-min time point. At the 30- and 45-min time points, 268 (15%) and 295 (17%) are associated with the +1 position. This information is now included in the text (Lines 267-270).

(2) I found it strange in the discussion and elsewhere that when mentioning the DNA

specificity of ATP-dependent chromatin remodelers that only RSC and CHD1 are mentioned. INO80, the paralog complex of SWR1, is probably one of the best complexes with DNA sequence specificity and is the most similar to SWR1. And yet for some reason is never mentioned in that context.

Agreed. We have revised the discussion in line 453-455 to include a mention of INO80 and have added the relevant reference.

Reviewer #3 (Remarks to the Author):

In this manuscript, the researchers explored the preference of the chromatin remodeler SWR for nucleosomes reconstituted with genomic DNA in budding yeast, particularly focusing on its deposition of the histone variant H2A.Z to replace H2A. Their findings indicated that SWR exhibits a preference for nucleosomes in the intergenic region rather than coding regions. Moreover, they demonstrated that the insertion of polyadenine into the nucleosome near the H2A-H2B binding sites stimulates the deposition of H2A.Z by SWR. The authors concluded that the genome encodes information influencing remodeler-mediated targeting of H2A.Z.

This study is innovative and holds significance for researchers in the chromatin field. Overall, the manuscript is well-crafted and is suitable for publication in Nature Communications.

We thank Reviewer 3 for appreciating the significance of our study.

The following issue needs to be addressed before the manuscript can be accepted for publication.

“SWR prefers to a nucleosome” can have different meanings. It could mean that SWR has a higher affinity towards a nucleosome or SWR catalyzes the exchange reaction on a nucleosome more efficiently, or both. Both can contribute to the in vivo observation.

Agreed. The usage of the word ‘prefer’ is intentional, as it is inclusive of the possibility that poly(dA)-enriched nucleosomes can increase affinity to and/or activity of SWR. To rigorously test these possibilities, one approach is to perform biochemical assays to determine the affinity of SWR towards recombinant nucleosomes with and without the poly(dA) track. For native nucleosome substrates, we can see if immobilized SWR can preferentially pull down a subpopulation of nucleosomes and identify their positions by sequencing. To see if poly(dA)-enriched nucleosomes can stimulate SWR activity, we can perform a single turnover assay or perform Michaelis-Menton analysis to determine k_{cat}. While these experiments are highly important, they are beyond the scope of the current study.

The authors measured exchange preference in vitro. ChIP-seq data of SWR should provide additional information on its association to nucleosomes. It is still possible that the apparent affinity of SWR to the nucleosome through direct or indirect interactions

determines the incorporation of H2A.Z in vivo. The authors proposed that "SWR acts upstream of RSC and INO80 to replace A-B dimers in nascent, promoter-proximal nucleosomes with Z-B dimers." Their model (Fig. 8) suggests that SWR is initially recruited to the NDR region, catalyzes the replacement reaction, and pushes the H2A-Z nucleosome to the +1 site. This implies that SWR should occupy the entire NDR, not necessarily close to the +1 site. Yet, existing data, such as Yen et al.'s study (Cell 2013. PMID: 24034248), contradicts the authors' conclusion, showing that SWR is close to the +1 site in vivo. To address this discrepancy, the authors are encouraged to reanalyze or obtain high-resolution SWR-ChIP-seq data to exclude other potential explanations.

Reviewer 3 is correct to point out that the ChIP-exo data in Yen et al.'s study showed that the SWR complex is predominantly mapped to the +1 site, whereas our Z-enrichment value determined by our in vitro experiment are more enriched upstream of +1 (Figure S8B). As mentioned in our response to Reviewers 1 and 2, and our revised discussion (Lines 426-429), one explanation for the failure of SWR to target many +1 nucleosomes under our reaction conditions is the lack of long DNA linker in our native nucleosome library that is the dominant recruiting mechanism of the +1 site.

However, it is important to note that many endogenous H2A.Z sites that require SWR activity (revealed by our SWR-dependent H2A.Z data in Figure 1), including those within the H2A.Z islands, has no detectable ChIP-exo Swr1 signal (Figure R2). This means that even though SWR has engaged and reacted with those nucleosomes within the H2A.Z islands, ChIP-exo is not sensitive enough to capture the transient nature of SWR's remodeling activity. In fact, the interaction of chromatin remodelers with their nucleosomal substrates is notoriously transient and difficult to ChIP, as demonstrated by Gelbart et al. in G&D 2005 PMID: 15833917. Therefore, the existing mapping data of SWR has limitation. We are actively investigating the mechanistic nature of SWR's preference to poly(dA:dT) containing nucleosomes, but this is beyond the scope of the current study. We have included a figure similar to R2 in Figure S3. However, we did not include the ChIP-exo data of Swr1, as the limitation of the ChIP-exo technique is not a main point of this study.

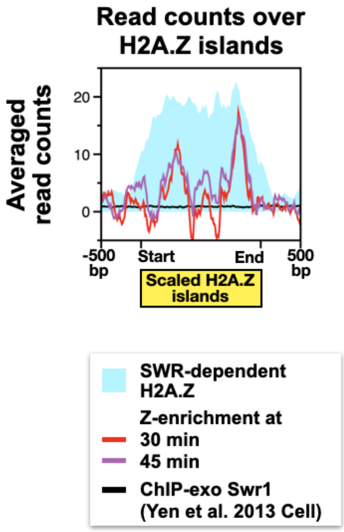

Figure R2: Average read counts were plotted along H2A.Z islands and flanking regions. The starts and ends of 264 H2A.Z islands (defined as having six or more consecutive H2A.Z sites) were aligned, and the associated Z-enrichment values, measuring the SWR's in vitro preference, along with the ChIP-exo data of Swr1 by Yen et al 2013 (SRA accession SRR948403) were plotted for comparison.
