# Supplementary material for: H2A.Z deposition by the SWR complex is stimulated by polyadenine DNA sequences in nucleosomes
Source: PLoS Biol. 2025 May 12;23(5):e3003059. doi: 10.1371/journal.pbio.3003059 (PMC12068740; doi:10.1371/journal.pbio.3003059)
Supplement: Attachment — Submitted filename: Respond_to_PLOS_REVIEWS_Oct_2024_v5.pdf [file pbio.3003059.s047.pdf]

REVIEWS:

Our responses to reviewers' comments are highlighted in blue.

Reviewer #1: The authors had done a good job of addressing many points raised previously, but some key concerns remain that have not been adequately answered. The question remains whether the DNA sequence effect observed for SWR complex removing H2A and replacing it with H2A.Z is due to increased affinity of SWR for nucleosomes and/or impacts the enzymatic activity. The requested experiments appear to be straight forward such as changing the enzymatic reactions from having 20X excess nucleosomes where SWR affinity can be a determining factor to where SWR1 is equal molar or in slight excess to ensure SWR's affinity is not a key factor. Given the title of the paper with its potential implications (i.e. targeting) and the importance of this determination, it seems imperative to perform the necessary experiments. Is there any experimental reason why these experiments cannot be performed? Even though these experiments do not address other questions which the authors point out such as what step in the enzymatic reaction; whether the rate at which the dimer is exchanged, the other dimer is inserted or the rate of ATP hydrolysis, these experiments are nonetheless essential for this paper and are the minimal that is required.

The other approach to address this question is to perform gel shift experiments or pull down assays with nucleosomes that have or don't have the poly dA-dT tracts as pointed out by the authors. It should be sufficient to perform these experiments with only the recombinant nucleosomes and not have to go the level of doing the same experiments with the native nucleosomes combined with whole genome sequencing. In this way it should be feasible for the authors to perform these limited number of experiments and avoid the concerns expressed by the authors of this being too expansive for the current paper. I don't feel this is beyond the scope of the paper but is rather critical information that is missing for the story to be considered complete.

We thank Reviewer 1 for encouraging us to investigate whether SWR's preference for nucleosomes containing poly(dA:dT) tracks is driven by affinity. Initially, we hesitated to perform the binding study because of the high concentration of SWR required for the experiment. However, we successfully concentrated our affinity-purified SWR, achieving a sufficient concentration to carry out the binding experiment. Following the reviewer's suggestion, we performed a gel shift experiment based on the protocol by Ranjan et al. (2015; DOI: 10.7554/eLife.06845) (Figure R1). Our new data demonstrate that SWR indeed exhibits a higher affinity for nucleosomes with a 10-bp poly(dA:dT) track inserted between SHL -4 and -5, which explains the enhanced activity for this substrate in our in vitro remodeling reaction. We have included the binding experiment in the revised Figure 6E-F & S12C and Lines 346-351 of the main text.

Figure R1 (Figure 6E-F and replicate in Figure S12C)

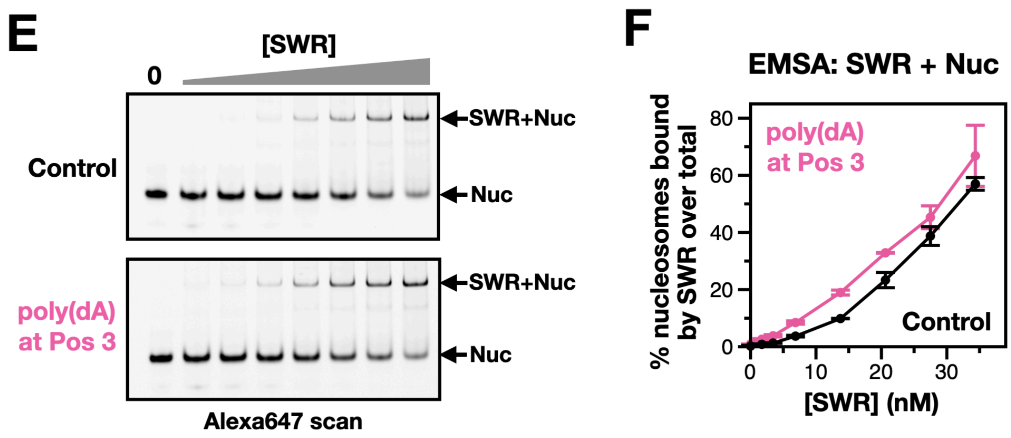

The authors' suggestion it is OK to not consider the effect of linker DNA on the DNA sequence specificity of the SWR complex because SWR can still do dimer exchange with nucleosomes that have no or only short linker DNA seems incomplete and potentially misleading. It is possible that the DNA sequence specificity they observe with nucleosomes lacking sufficient linker DNA may not be observed when nucleosomes have sufficient linker DNA length. In the event this is the case then it would show this DNA sequence specificity is very likely not biologically relevant. We agree with the authors' comments about the potential for a complex interplay between linker DNA, DNA sequence specificity and histone modifications, which in its totality is beyond the scope of this paper. The linker DNA experiments however only need be done with the model mononucleosome substrates as already performed with the simple change of adding on linker DNA and not have to perform the myriad of other potential experiments that could be done towards this general purpose. It doesn't seem like a good rationale to separate out the impact of linker DNA from the DNA sequence of the core nucleosome as suggested in the paper given the likely interplay between the two.

The reviewer's point is well taken. In response, we conducted the suggested in vitro experiment. We generated mono-nucleosomes with a 50-bp linker on one end, which is optimal for SWR binding (doi.org/10.1016/j.cell.2013.08.005), and a 7-bp linker on the other end (Figure R2, which is the new Figure 8). The control nucleosomal substrate consisted of the Widom DNA with canonical histones, and the experimental dA_{10} substrate contained a 10-bp poly(dA:dT) track inserted between SHL -4 and -5 (Figure R2, yellow region). Upon incubation with SWR, the control AA nucleosomes were converted into the heterotypic AZ and ZA species (Figure R2B-C), migrating as a doublet in the native gel, before progressing to the ZZ product (Figure R2D-E). Previously, we showed that the AZ species (H2A.Z distal to the long linker) corresponds to the lower band of the doublet, while the ZA species (H2A.Z proximal to the long linker) is the upper band (Sun et al. 2020; doi.org/10.1016/j.celrep.2019.12.006). This difference in migration is caused by the asymmetry of the long linker relative to the position of FLAG tag on the Z-B dimer, and we have previously confirmed this by site-directed hydroxyl radical cutting (Sun et al. 2020).

Figure R2 (Figure 7)

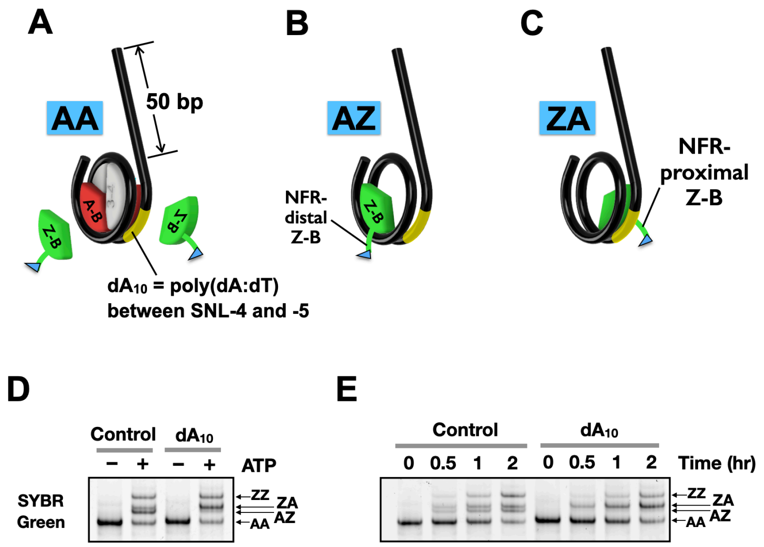

Consistent with a stimulatory effect of the poly(dA:dT) track on SWR's activity, SWR exhibited a strong insertion preference toward the side of the nucleosome with the poly(dA:dT) track, as indicated by the more intense upper ZA band in the AZ/ZA doublet (Figure R2D-E). Notably, the increased H2A.Z insertion on the ZA side in the experimental dA_{10} substrate was accompanied by a decreased H2A.Z insertion on the AZ side without the poly(dA:dT) relative to control (Figure R2D-E). This suggests that SWR has redistributed the insertion of H2A.Z favorably towards the side with the poly(dA:dT) sequence, consistent with our model. The new data is presented in revised Figure 7 and Lines 346-368 of the main text.

Reviewer #2: The goal of this study is to characterize the DNA sequence determinants that stimulate the H2A/H2A.Z exchange activity of the SWR complex and its targeting to specific nucleosomes in the yeast genome (namely +1 nucleosomes at the transcription start site of most yeast genes). The question is relevant to the field of chromatin biology and the experiments are well designed with a clever combination of in vivo and in vitro approaches. They conclude that the "preferred" nucleosome substrates for SWR activity contain poly A tracts upstream of the dyad axis and propose a model in which SWR acts on nucleosomes initially assembled in the NDR or the promoter region before remodeling steps mediated by RSC and Ino80 that re-position these nucleosomes to their "canonical" +1 position. While the model seems plausible based on their results, there is a lack of crucial controls, which coupled with some puzzling reasoning undermines the robustness of their conclusions and their interpretation of the data as I explain below.

We thank Reviewer 2 for recognizing our well-designed experiments and our innovative in vivo and in vitro approaches, as well as the importance of the question posed in the paper. We also understand the reviewer's concerns regarding certain conclusions in our paper. In

the following sections, we will address these concerns, either through the addition of control experiments and analysis or by adjusting our conclusions.

1. There are logical flaws in the interpretation of their otherwise ingenious and informative in vivo crosslinking approach from Figure 1B. Just because H2A.Z incorporation is reduced by ~50% in swc2D mutants compared to wt, it does not mean that H2A.Z is incorporated by SWR independent mechanisms in 50% of all H2A.Z nucleosomes in wt cells. It just means that H2A.Z incorporation in the absence of SWR activity is possible but incorporation efficiency is two fold lower than in wt where SWR activity is normal. This assay does not exclude the possibility that H2A.Z incorporation is SWR dependent for all H2A.Z containing nucleosomes in WT cells.

The reviewer's point is well taken. We have removed all claims that H2A.Z is deposited by SWR-independent mechanism in the *swc2Δ* mutant.

2. Despite their claims to the contrary, that conclusion (that H2A.Z is incorporated by SWR independent mechanisms in 50% of all H2A.Z nucleosomes in wt cells) is not corroborated by their H2A.Z ChIP-seq experiments in wt and swc2D cells (Figure 1 E-G). Even though they do not explain very well how they normalized the data, I'm assuming each dataset (wt and swc2D) was normalized separately to its respective total read count (ppm) and then "corrected" for the differences in H2A.Z incorporation between wt and swc2D by dividing the swc2D signal by 0.558 (or multiplying the wt signal by the same factor). Incidentally, the latter correction is quite unnecessary since the first normalization to total read count already gives enrichment peaks relative to the rest of the genome for each dataset. This correction does not actually allow for a direct comparison between equivalent genomic loci in each dataset, because each dataset was internally normalized first. One can only make direct comparisons between two different datasets if they are externally normalized to the same "spike-in" control.

Despite this confusing normalization method, they do however reach the correct conclusion that, since H2A.Z enrichment levels in swc2D are indistinguishable from background, SWR is responsible for all H2A.Z incorporation into specific nucleosome locations (+1, -1, what they call H2A.Z islands etc..).

We agree with Reviewer 2 that H2A.Z enrichment levels in *swc2Δ* are indistinguishable from background and that SWR is responsible for all distinct H2A.Z sites in yeast. We also agree that including a spike-in control would provide the most robust approach for comparing different datasets. However, implementing an appropriate spike-in control in this context will require significant technical development, which will exceed the time constraints for resubmission. I will further elaborate our reasoning below.

Since our statistical analysis (lines 770-782) indicates that the SWR-dependent H2A.Z sites identified through our current approach are robust, and the Reviewer concurs with our

conclusion that the identified H2A.Z nucleosomes are dependent on SWR activity, we respectfully propose to retain our current analysis.

To perform the H2A.Z IP experiment in our study, we first extracted crude chromatin from spheroplasts, digested the chromatin with MNase to obtain predominantly mononucleosomes (Figure S1C), quantitatively pulled down H2A.Z-containing nucleosomes using anti-FLAG agarose targeting the FLAG tagged H2A.Z (Figure S1D), and eluted the H2A.Z with FLAG peptides.

One type of spike-in control suitable for this strategy involves adding recombinant FLAG-tagged nucleosomes containing known DNA sequences into fixed amounts of mononucleosome samples after the MNase digestion step. However, the accuracy of this spike-in is strongly influenced by variability in MNase digestion efficiency and variability in DNA quantification of the MNase-digested samples, which are heavily contaminated with RNA, thereby requiring additional DNA purification, which further introduces variability due to DNA loss.

A potentially better alternative would be to use a different yeast species, such as *S. pombe*, which expresses a FLAG tagged histone, such as Pht1 (H2A.Z). In this approach, we could quantify our *S. cerevisiae* input samples cell counting and adding a known amount of *S. pombe* cells as spike-in control. However, this approach will require strain construction and optimization of our glucanase digestion protocol. Given the time constrain for resubmission and the low likelihood that the spike-in control would alter our conclusions, we have prioritized addressing other, more pressing questions.

Unfortunately, they then go a step too far and over interpret their data by claiming that 50% of wt H2A.Z nucleosomes do not depend on SWR activity. Their data only suggests that H2A.Z is incorporated randomly in the absence of SWR activity. There is however no evidence in their data that this kind of random SWR-independent H2A.Z incorporation also occurs in wt cells where SWR activity is normal. Precisely because the H2A.Z ChIP signal in swc2D is indistinguishable from the background, it is impossible to tell what proportion of that signal actually comes from bona fide H2A.Z nucleosomes and what proportion comes from non-specific antibody binding to random epitopes. The latter would be all the more likely if H2A.Z incorporation is reduced genome-wide, as they show in Figure 1B. This is why ChIP-seq data are, as a rule, normalized to input, which had apparently not been done for this analysis. Consequently, the identification of SWR dependent H2A.Z nucleosomes in wt cells by subtraction of swc2D data from wt data amounts to the same as normalization of wt data to its own input. The second is actually more accurate since nucleosome positions in wt and swc2D may vary (another control that is not provided). Nevertheless, I do agree that the H2A.Z nucleosomes that they identified as SWR dependent in wt are indeed dependent on SWR activity but they should really use inputs for normalization, eliminate unnecessary normalization "corrections" and take out their conclusion about SWR-independent H2A.Z incorporation in wt cells.

To assess whether loss of *SWC2* affects nucleosome positioning, we plotted the sequencing reads from the input of WT and *swc2Δ* around +1 nucleosome dyads (Figure R3). We observe no major differences in nucleosome positioning, suggesting that our normalization procedure—subtracting the H2A.Z enrichment signals of *swc2Δ* from those of WT—is valid.

Figure R3 (Figure S17)

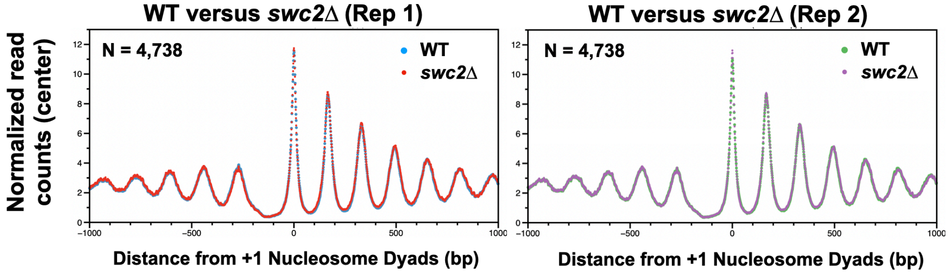

One disadvantage of normalizing the H2A.Z IP signal over input by division is that it amplifies signal variance when the denominator (input signal) is small. As a result, H2A.Z signals in regions with lower nucleosome occupancy or fuzzier positioning, such as genic regions, become less reliable compared to well-positioned regions, such as +1 nucleosomes. By contrast, normalizing the WT H2A.Z IP signal by subtraction the *swc2Δ*-derived background avoids this amplification of variance in low-occupancy or fuzzy regions, making it a better approach in this situation.

Although we retain our current analysis, we have provided the sequencing reads from the input and flow-through fractions of our IP reactions for transparency and independent validation. The data has been uploaded to SRA and GEO under the accession numbers PRJNA1060626 and GSE252824, respectively.

3. Their strategy from here on is to identify nucleosomal DNA sequence features that favor SWR activity with an in vitro assay in which SWR incorporates H2A.Z into native mononucleosomes derived from htzD/swrD cells. While the strategy is reasonable, there are two important controls that are missing and without which their results are difficult to interpret correctly.

The results in Figure 5B-C and S8B show a puzzling enrichment of H2A.Z nucleosomes in regions that are depleted of nucleosomes in vivo: NDRs in promoters and at the 3' end of genes. As pointed out by reviewer 1 (from the previous review of this article), these sequences should be depleted from their set of native mononucleosomal substrates if a) their MNase digestion degraded all internucleosomal DNA and left only mononucleosomes with very short 6/7 bp linkers, and b) nucleosome positioning in their htzDswrD strain from which they derived their mononucleosomal set is identical to the wt nucleosome

positioning of their native H2A.Z reference dataset.

To control for a) and exclude the possibility that +1 nucleosomes from their native mononucleosome set are favored by SWR just because they have longer linkers, they need to provide fragment size distribution of their paired-end reads, as was suggested by reviewer 1. Incidentally their response to reviewer 1 does not address the problem: just because one expects that AT rich regions will be preferentially cut by MNAse does not mean that was actually the case in their mononucleosome set (MNAse digestions are finicky and it's quite easy to over or under-digest), especially since judging from the smear in lanes 4-6 of Figure 3B they almost certainly isolated mononucleosomes with linkers of variable sizes.

We thank Reviewer 2 for pointing out that the native nucleosomal substrates used to interrogate SWR preferences might have variable linker lengths, contributing to SWR's substrate selectivity. In response, we analyzed the read lengths of our native nucleosomes and observed a broad distribution of linker lengths. Indeed, SWR showed a preference for nucleosomes with longer linkers, as evidenced by the higher proportion of longer DNA fragments, especially >155 bp, in the Eluate fraction (H2A.Z-containing) compared to the FT fraction (unreacted nucleosomes) (Figure R4, left).

Figure R4 (Figure S16)

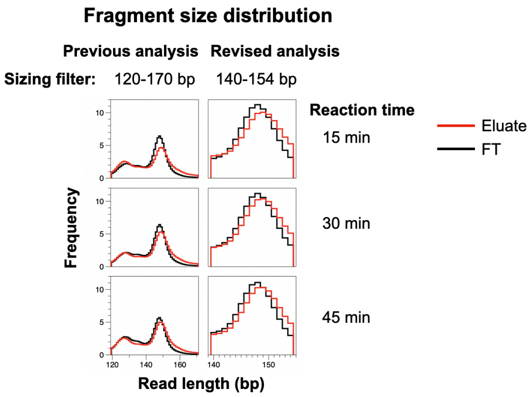

To reduce the influence of linker length on SWR's selectivity in our assay, we applied a more stringent sizing filter (140-155 bp) during data analysis (Figure R4, right). After re-ranking SWR-preferred nucleosomes, the previously puzzling H2A.Z enrichment in the inter-nucleosomal regions was largely resolved (Figure R5).

Figure R5 (Figure 4A-B)

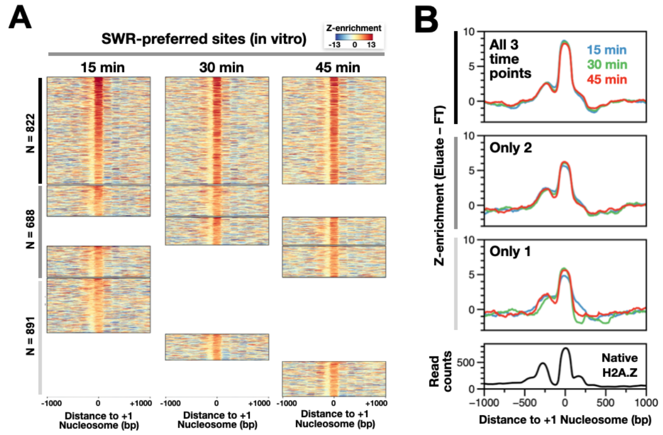

Notable, when the new list of SWR-preferred nucleosomes was used a higher percentage of these nucleosomes overlap with endogenous H2A.Z sites. At the 15-, 30, and 45-min time points, 35%, 41%, and 45% of preferred nucleosomes overlap with endogenous H2A.Z sites, as opposed to 32%, 39%, and 44% in our previous analysis. Therefore, we are extremely grateful for the Reviewer for this insightful suggestion. Figures 3-5, S7, S8, S10, and S11 were replotted using data processed with the stringent sizing filter. The text corresponding to these revised figures can be found in lines 236-314.

To control for b) they need to provide nucleosome positioning data for their htzDswrD strain, again something that was suggested by reviewer 1 and was not addressed in their response. Indeed, a potential increase in nucleosome occupancy in the NDRs coupled with "fuzzy" positioning in ORFs in that strain would essentially enrich for NDR sequences in their substrate set, in which case the apparent preference of SWR for polyA rich NDR regions could just be due to an over-representation in their substrate set of what would have been wt NDR sequences but are actually nucleosomal sequences in their htzDswrD mutant. It is impossible to make any meaningful conclusions about DNA sequence preferences of SWR targets without these two controls.

To assess if *htz1Δswr1Δ* mutants are defective in nucleosome positioning, we performed MNase-seq on WT and *htz1Δswr1Δ* cells and plotted the read center counts around the +1 nucleosome dyads. We detected no observable changes in nucleosome positioning in the *htz1Δswr1Δ* mutant relative to WT, indicating that the loss of H2A.Z (Htz1) and Swr1 does not alter nucleosome positioning (Figure R6A).

Figure R6 (Figure S17)

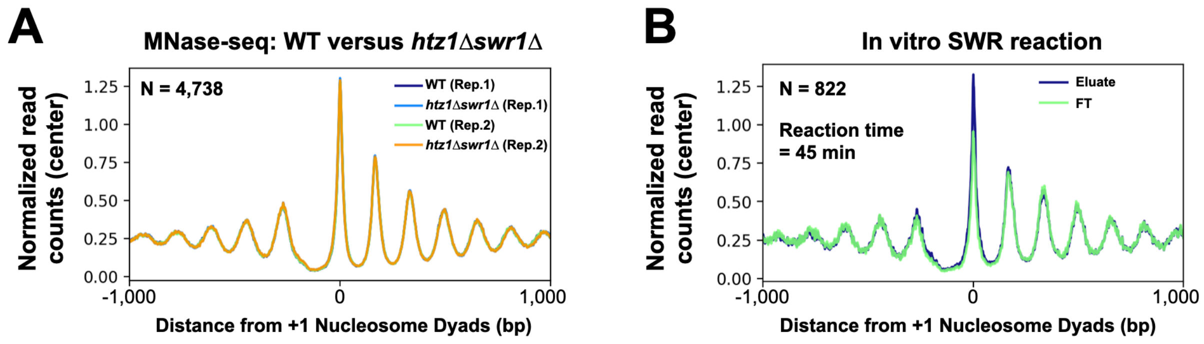

To evaluate if SWR preferentially reacts with mispositioned nucleosomes under our reaction conditions, we plotted the read center counts of nucleosomes from the eluate fraction (preferred) and the FT fraction (unpreferred) from the SWR-mediated reaction (at 45 min) (Figure R6B). The data again shows no observable changes in nucleosome positioning, indicating that SWR does not preferentially target mispositioned nucleosomes. Similarly, the eluate and FT fractions at the 15- and 30-min reaction time points exhibited no observable change in nucleosome positioning (not shown). These control plots can be found in Figure S17 and are described in lines 768-774 of the main text.

4. The analysis in Figure 6 shows a modest 25% enrichment in dAdA frequencies in the first half of what they call SWR "preferred" nucleosomes, which represent only 3% of all isolated H2A.Z nucleosomes and have at best only a twofold enrichment over flow through (see Figure 4 and Figure 3). I would advise the authors to be very cautious when they draw general conclusions on SWR activity based on these modest sequence preferences. The analysis shows oscillations in dAdA frequencies over the first half of a composite nucleosome based on 1700 nucleosome sequences. It is not clear how many if any of these nucleosomes actually have 10 A or 13 A tracts in the first half of their DNA sequence. The test constructs from Figure 7 with 10 A and 13 A tracts at different positions within the first half of the nucleosome are therefore hardly representative of any one nucleosome from the "preferred" SWR set. While they do show that test substrates with a polyA tract at positions 2 and 3 are modestly "preferred" by SWR over a control substrate (35% to 46%), a preference probably due to the inherent instability of nucleosomes with this type of sequence, it is difficult to assess the in vivo frequency of nucleosome formation over these kinds of sequences and consequently estimate how general the model in Figure 8 may be, if it happens at all.

To address the modest enrichment of dAdA frequencies observed in the preferred SWR nucleosomes, we reanalyzed the data by: (1) requiring enrichment at two or more time points; and (2) including unpreferred nucleosomes on the same graph for direct comparison. The updated figure demonstrates a more pronounced left-side enrichment of dAdA frequences and right-side enrichment of dTdT (Figure R7). Additionally, the opposite

pattern observed in the unpreferred sites independently confirms SWR's substrate preference.

Figure R7 (Figure 5)

Old figure 6

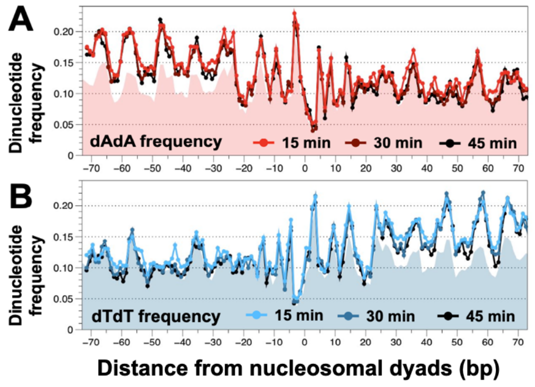

New Figure 5

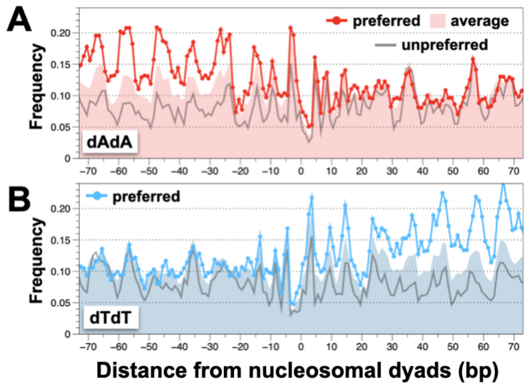

To evaluate whether the preferred nucleosomes contain longer dA or dT tracks, we plotted the frequences of five consecutive dA (dA_5) and dT (dT_5) residues (Figure R8). Consistent with the results of the dinucleotide motif analysis, the dA_5 and dT_5 frequencies exhibited a symmetrical dA:dT enrichment profile relative to nucleosome averages. This further supports the conclusion that SWR preferentially remodels nucleosomes containing dA:dT tracks. The revised plots can be found in Figure 5 and are described in Lines 284-300.

Figure R8 (Figure S11)

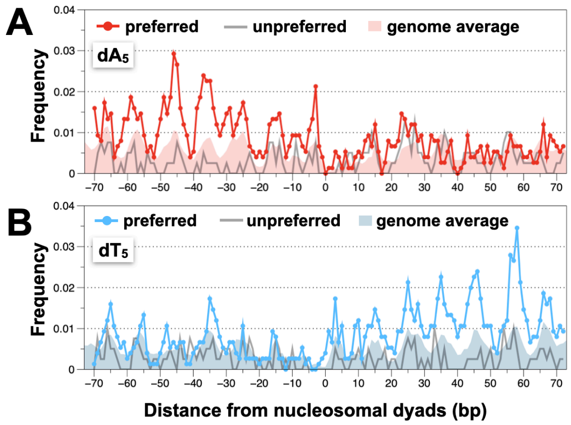

We chose dA_5 and dT_5 instead of longer dA/dT tracks because, while there are preferred nucleosomes with dA_{10} and dT_{10}, many longer tracks were interrupted by single or short non-dA or non-dT residues. Our lists of SWR-preferred and unpreferred nucleosomes,

along with their sequences, are included in Supplemental Data File 1 for readers' reference.

To address how many SWR-preferred nucleosomes contain poly(dA:dT) tracks, we found that **79%** of preferred nucleosomes (N = 1,069) contain one or more dA_5 or dT_5, compared to **28%** of unpreferred nucleosomes (N = 1,236). As a reference, **57%** of nucleosomes across the genome (N = 67,534) contain one or more dA_5 or dT_5. On average, SWR-preferred nucleosomes contain **3.2** dA_5 or dT_5 sites, while unpreferred nucleosomes contain **0.46** site, and genome-wide nucleosomes contain **1.5** sites. Regarding uninterrupted dA_{10} or dT_{10} sites, **9.0%** of preferred nucleosomes contain these sequences, compared to **0.16%** of unpreferred nucleosomes and **2.3%** of genome-wide nucleosomes. The new plots can be found in Figure S11 and are described in Lines 302-314.

Designing an in vitro substrate that fully represents in vivo conditions is challenging, as in vivo nucleosomal substrates are inherently diverse. The goal of the in vitro experiments shown in Figure 6, S12, and 7 is to test the hypothesis that the presence of a poly(dA:dT) track is sufficient to enhance SWR's activity. Although the observed stimulatory effect is modest, the data is highly reproducible (Figure 6, S12, and 7).

In cells, multiple factors influence SWR-mediated H2A.Z deposition, including NFRs, histone acetylation, and, as revealed by this study, specific DNA sequences. Preferred DNA sequences may destabilize nucleosomes; however, nucleosomes across the genome are biochemically and structurally diverse. This diversity arises from their association with distinct DNA sequences, incorporation of histone modifications and/or histone variants, and the temporal status of chromatin maturation (e.g., nascent versus mature nucleosomes), all of which affect nucleosome stability. Therefore, nucleosome instability *is* an integral feature of chromatin biology.

Traditional in vitro assay utilizing synthetic substrates, such as nucleosomes reconstituted with the Widom 601 sequence, often fail to capture the complexities of native nucleosome substrates. This study is the first to investigate SWR-mediated H2A.Z deposition while accounting for the intrinsic diversity of nucleosomal substrates. The hundreds of sequences identified here are potential tools for biochemical and structural studies to elucidate SWR's remodeling mechanism. By addressing the inherent complexity of nucleosomal substrates, this study provides a more nuanced understanding of the mechanism underlying chromatin remodeling reactions.
